# The nuclear receptor REV-ERBα is implicated in the alteration of β-cell autophagy and survival under diabetogenic conditions

**DOI:** 10.1038/s41419-022-04767-z

**Published:** 2022-04-15

**Authors:** Matthew R. Brown, Damien Laouteouet, Morgane Delobel, Orianne Villard, Christophe Broca, Gyslaine Bertrand, Anne Wojtusciszyn, Stéphane Dalle, Magalie A. Ravier, Aleksey V. Matveyenko, Safia Costes

**Affiliations:** 1 Department of Physiology and Biomedical Engineering, Mayo Clinic School of Medicine, Mayo Clinic, Rochester, MN USA; 2 Institute of Functional Genomics, University of Montpellier, CNRS, INSERM, Montpellier, France; 3 Laboratory of Cell Therapy for Diabetes (LTCD), PRIMS facility, Institute for Regenerative Medicine and Biotherapy (IRMB), University hospital of Montpellier, Montpellier, France; 4 Department of Endocrinology, Diabetes, and Nutrition, University Hospital of Montpellier, Montpellier, France; 5 Division of Endocrinology, Metabolism, Diabetes, and Nutrition, Department of Medicine, Mayo Clinic, Rochester, MN USA

**Keywords:** Macroautophagy, Diabetes

## Abstract

Pancreatic β-cell failure in type 2 diabetes mellitus (T2DM) is associated with impaired regulation of autophagy which controls β-cell development, function, and survival through clearance of misfolded proteins and damaged organelles. However, the mechanisms responsible for defective autophagy in T2DM β-cells remain unknown. Since recent studies identified circadian clock transcriptional repressor REV-ERBα as a novel regulator of autophagy in cancer, in this study we set out to test whether REV-ERBα-mediated inhibition of autophagy contributes to the β-cell failure in T2DM. Our study provides evidence that common diabetogenic stressors (e.g., glucotoxicity and cytokine-mediated inflammation) augment β-cell REV-ERBα expression and impair β-cell autophagy and survival. Notably, pharmacological activation of REV-ERBα was shown to phenocopy effects of diabetogenic stressors on the β-cell through inhibition of autophagic flux, survival, and insulin secretion. In contrast, negative modulation of REV-ERBα was shown to provide partial protection from inflammation and glucotoxicity-induced β-cell failure. Finally, using bioinformatic approaches, we provide further supporting evidence for augmented REV-ERBα activity in T2DM human islets associated with impaired transcriptional regulation of autophagy and protein degradation pathways. In conclusion, our study reveals a previously unexplored causative relationship between REV-ERBα expression, inhibition of autophagy, and β-cell failure in T2DM.

## Introduction

Type 2 diabetes mellitus (T2DM) is a major public health problem with more than 400 million people affected in the world. This chronic metabolic disorder is characterized by hyperglycemia secondary to the decline of β-cell secretory function and mass (e.g., β-cell failure) and is usually accompanied by a reduced sensitivity to insulin. The β-cell functional failure and cell deficit in T2DM are attributed in part to β-cell’s exposure to a variety of intracellular and extracellular pro-diabetogenic stressors which include (but not limited to) proteotoxicity, chronic hyperglycemia and hyperlipidemia, and/or low-grade inflammation. Although these diabetogenic stressors promote β-cell failure in T2DM with some mechanistic differences, they commonly appear to alter the protein degradation pathway macroautophagy (hereafter referred to as autophagy) thus contributing to the induction of β-cell dysfunction and apoptosis [1–5].

Indeed, long-lived post-mitotic secretory cells, such as β-cells, bear a high burden of protein synthesis and folding and as such require robust protein quality control machinery for cellular function and survival [6]. Thus, autophagy plays a key role in the clearance of misfolded/ubiquitinated proteins, damaged organelles, and oligomerization-prone proteins to sustain intracellular homeostasis and metabolic functions [7]. In this process, cytosolic components are engulfed by autophagosomes that fuse with lysosomes to allow the degradation of the contents by lysosomal enzymes. Importantly, lack of β-cell autophagy leads to diabetes in transgenic mice [8–10] and altered autophagy is evidenced in β-cells of humans with T2DM [11]. Despite increasing evidence that impaired autophagy contributes to the pathophysiology of β-cell failure in T2DM, the mechanism responsible for defective autophagy is not fully understood.

Accumulating evidence points to the importance of the circadian clock for the proper regulation of β-cell secretory function, β-cell response to diabetogenic stressors, and β-cell survival and proliferation [12, 13]. The intracellular molecular clock machinery is comprised of key transcriptional activators CLOCK and its heterodimeric partner BMAL1 (encoded by *ARNTL*), and repressor genes that encode period (PER1,2) and Cryptochrome (CRY1,2) proteins [14]. Secondary regulatory loops involving nuclear receptors REV-ERBα/β (encoded by *NR1D1* and *2*) and RORα/β (encoded by *NR1F1* and *2*) provide molecular stabilization by acting as respective repressors and activators of *BMAL1* [15] and also play a role in the integration of the circadian clock with key cellular processes such as substrate metabolism, mitochondrial function, and differentiation [16]. Most notably, recent studies have revealed a novel role for the nuclear receptor REV-ERBα in the regulation of autophagy [17, 18]. Consistent with its role as a transcriptional repressor, REV-ERBα was recently shown to negatively regulate autophagy flux through transcriptional repression of key genes involved in vesicle nucleation, autophagosomal formation, lysosomal function, and mitophagy [18]. Correspondingly, pharmacological activation of REV-ERBα has been reported to inhibit autophagy and induce apoptosis in cancer cells [17], impair insulin secretion, and promote cell apoptosis in β-cell lines [19].

Whereas REV-ERBα expression was recently reported to be increased in β-cells in response to diabetogenic stressors (e.g., pro-inflammatory cytokines) [20], its role in autophagy regulation and potential contribution to β-cell dysfunction and apoptosis remains to be established. Therefore, in this study we set out to address whether REV-ERBα-mediated inhibition of autophagy caused by diabetogenic stressors contributes to the pathogenesis of β-cell failure. Our results demonstrate that glucotoxicity- and cytokine-induced β-cell deficit is attributed in part to REV-ERBα-mediated inhibition of autophagy. Importantly, negative modulation of REV-ERBα expression and activity provided protection from β-cell failure thus implicating REV-ERBα as a potential novel therapeutic target in T2DM.

## Results

### Pro-diabetogenic stressors augment REV-ERBα and p62 protein expression in β-cells

**Fig. 1:**
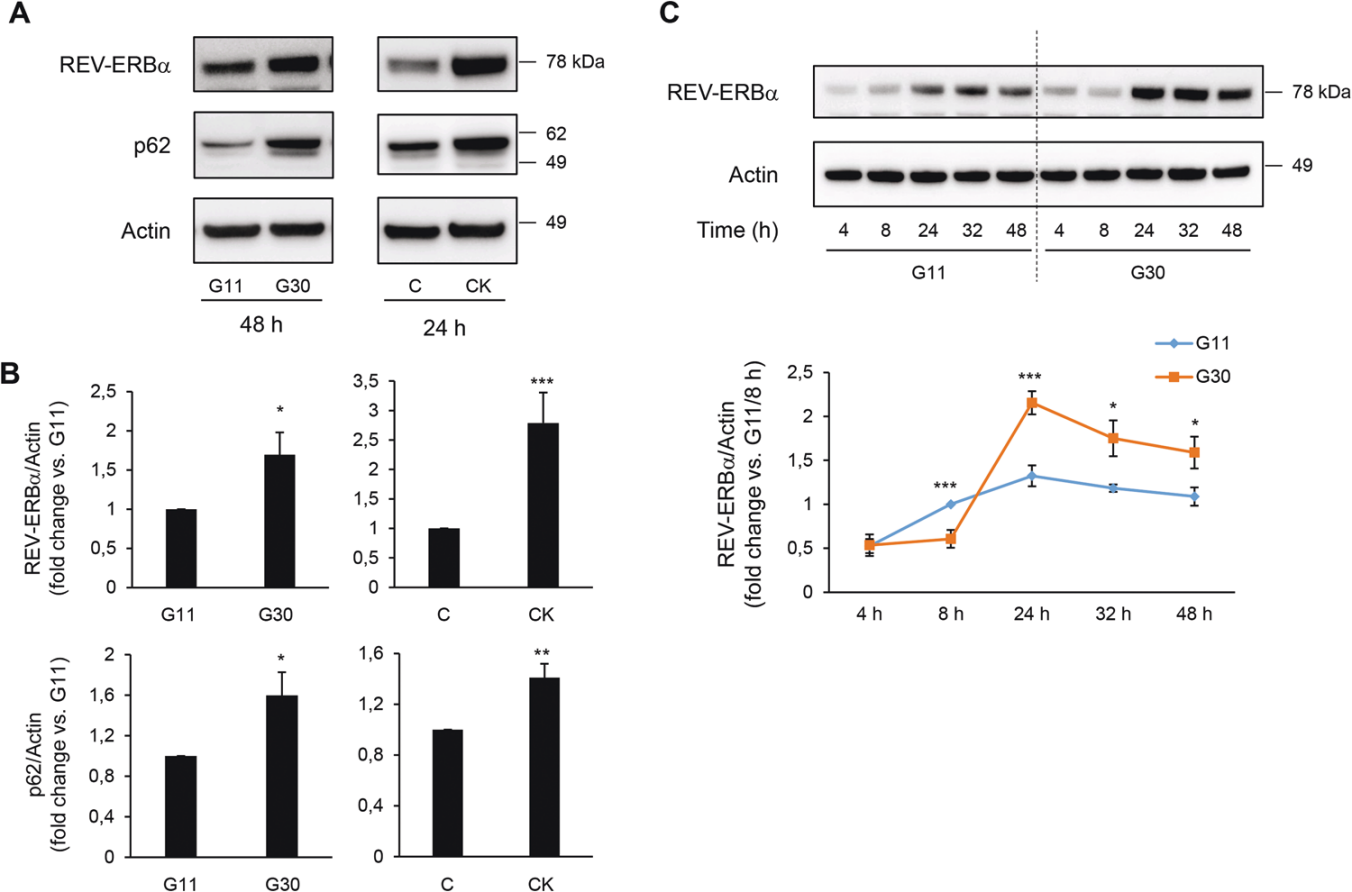
Pro-diabetogenic stressors augment REV-ERBα and p62 protein expression in β-cells. **A**, **B** REV-ERBα and p62 protein levels in INS-1E cells exposed to glucotoxicity for 48 h [30 mM glucose (G30) vs. 11 mM glucose as control (G11)], or pro-inflammatory cytomix [CK: 10 U/ml IL-1*β* (0.2 ng/ml), 500 U/ml TNF-*α* (50 ng/ml) and 100 U/ml IFN-*γ* (33 ng/ml)] vs. control (C, 11 mM glucose) for 24 h (*n* = 5 independent experiments). Graphs represent the quantification of the blots. **C** Temporal profile of REV-ERBα expression in INS-1E cells following 48 h exposure to G30 or G11 (*n* = 4–6 independent experiments). For all experiments, actin levels are used as loading control. Data are expressed as mean ± SEM; *P < 0.05, **P < 0.01, ***P < 0.001.

We first investigated whether conditions associated with T2DM lead to an increased expression of REV-ERBα and impaired autophagic flux. Exposure of INS-1E cells to high glucose concentration to mimic chronic hyperglycemia (30 mM glucose for 48 h) or pro-inflammatory conditions (cytomix: 10 U/ml IL-1*β*, 500 U/ml TNF-*α*, and 100 U/ml IFN-*γ* for 24 h) led to a ~2-3 fold increase in REV-ERBα protein expression (Fig. 1A, B, *P* < 0.05 and *P* < 0.001). Importantly, augmentation of REV-ERBα expression was accompanied by increased protein levels of p62, a well-defined marker of impaired autophagic flux previously shown to be increased in β-cells from T2DM patients [5, 10, 21]. To extend our findings, we next assessed the temporal profile of REV-ERBα expression in INS-1E cells following 48 h exposure to high glucose (Fig. 1C). Interestingly, whereas acute exposure to high glucose appeared to modestly attenuate REV-ERBα levels (*P* < 0.001 at 8 h post glucose administration), chronic high glucose led to robust upregulation of REV-ERBα (Fig. 1C, *P* < 0.001 and *P* < 0.05 at 24–48 h). Notably, these results are consistent with purported pro-survival and autophagy-stimulating effects of acute hyperglycemia and pro-apoptotic and anti-autophagic effects of chronic hyperglycemia (i.e., glucotoxicity) [22].

### Pharmacological activation of REV-ERBα inhibits β-cell autophagy and induces β-cell apoptosis

**Fig. 2:**
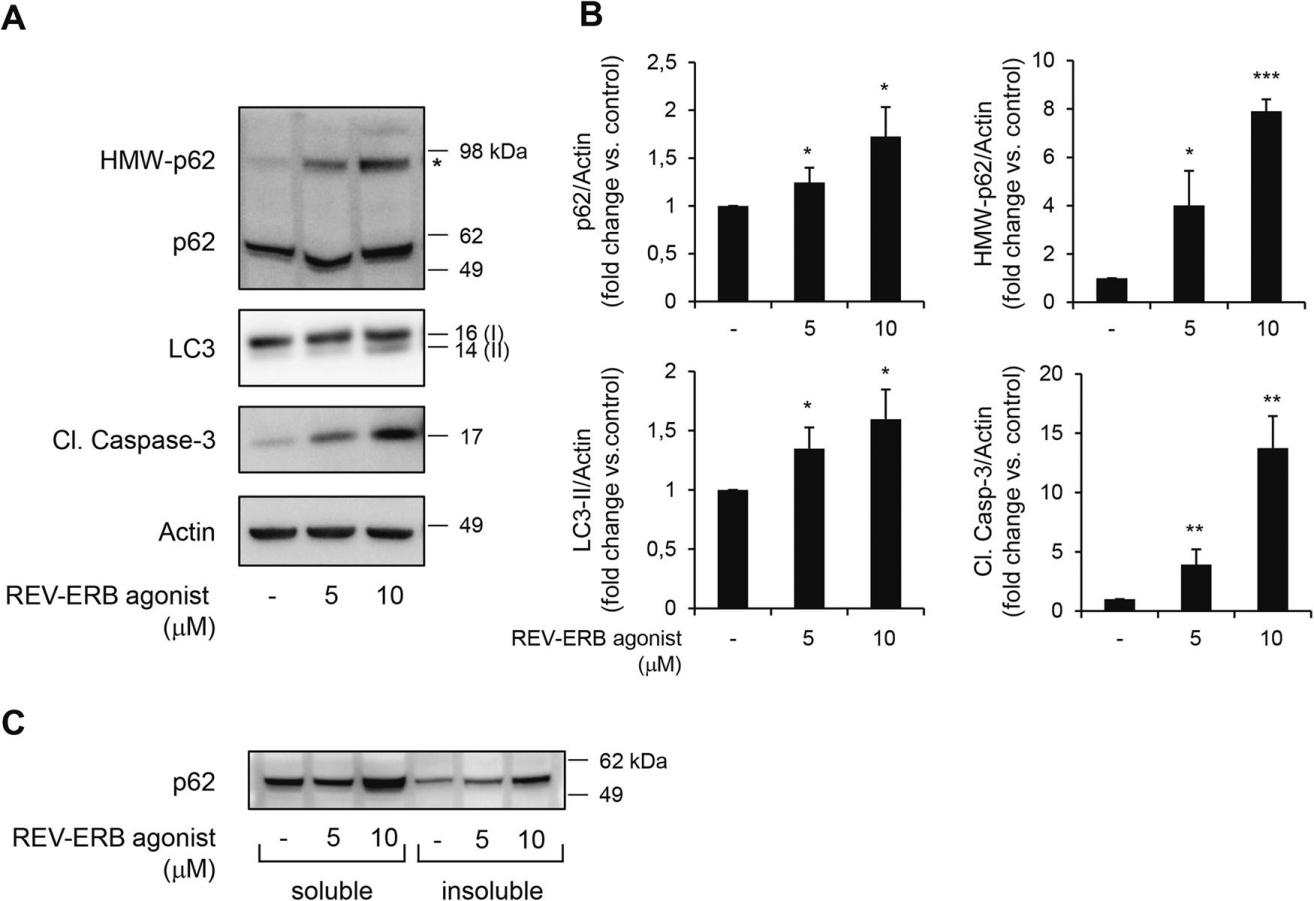
REV-ERBα agonism inhibits β-cell autophagy and induces β-cell apoptosis. **A** INS-1E cells were exposed to REV-ERB agonist (SR9009, 5 or 10 μM, 24 h) or control (vehicle DMSO). Protein levels of p62, high molecular weight (HMW)-p62, LC3, Cleaved Caspase-3, and actin (loading control) were analyzed by western blot. **B** Graphs represent the quantification of the blots (*n* = 4–5 independent experiments). Data are expressed as mean ± SEM; *P < 0.05, **P < 0.01, ***P < 0.001 vs. control. **C** Soluble and insoluble fractions obtained from INS-1E cells exposed to REV-ERB agonist (SR9009, 24 h) or control (DMSO). Protein levels of p62 were analyzed by western blot.

To evaluate whether REV-ERBα is directly involved in the regulation of β-cell autophagy and survival, we first employed pharmacological activation of REV-ERBα by treating β-cells with a well-characterized synthetic ligand SR9009 [17, 18, 23–25]. SR9009 ligand has been shown to directly bind REV-ERBα and enhance its repressor activity by facilitating the binding of nuclear receptor co-repressor-1 (NCoR) [17, 18, 23–26]. Our results demonstrate that exposure of INS-1E cells to SR9009 led to a robust dose-dependent increase in Cleaved Caspase-3 expression, a marker of apoptosis (Fig. 2A, B). In addition, exposure to SR9009 led to a dose-dependent increase in LC3-II levels (Fig. 2A, B), indicating an increased number of autophagosomes, which could be because of either induction of autophagy or decreased flux due to a blockade in fusion or degradation. To resolve this issue, levels of the autophagic substrate and adaptor p62 were examined: p62 protein levels were increased under SR9009 treatment (Fig. 2A, B) thus revealing that pharmacological activation of REV-ERBα decreases autophagy flux. Previous studies noted that p62 not only assists autophagic degradation of proteins but also, due to its polymeric nature, mediates its own aggregation when lysosomal degradation is limited [4, 27]. Interestingly, we noted the presence of a p62 high molecular weight band (98 kDa) in INS-1E cells exposed to SR9009 (Fig. 2A, B). This band reflects the presence of tightly aggregated forms of p62 [28] commonly detected in autophagy-deficient β-cells [10]. Since p62 aggregates are mostly insoluble, we next investigated whether p62 appears in the insoluble fraction of INS-1E cells exposed to SR9009. To proceed, we separated the detergent-soluble and -insoluble compartments by fractionation of cell lysates. We observed an increase in the levels of p62 in both the soluble and insoluble fractions following exposure to SR9009 (Fig. 2C), further supporting our observation of impaired lysosomal degradation following pharmacological activation of REV-ERBα.

**Fig. 3:**
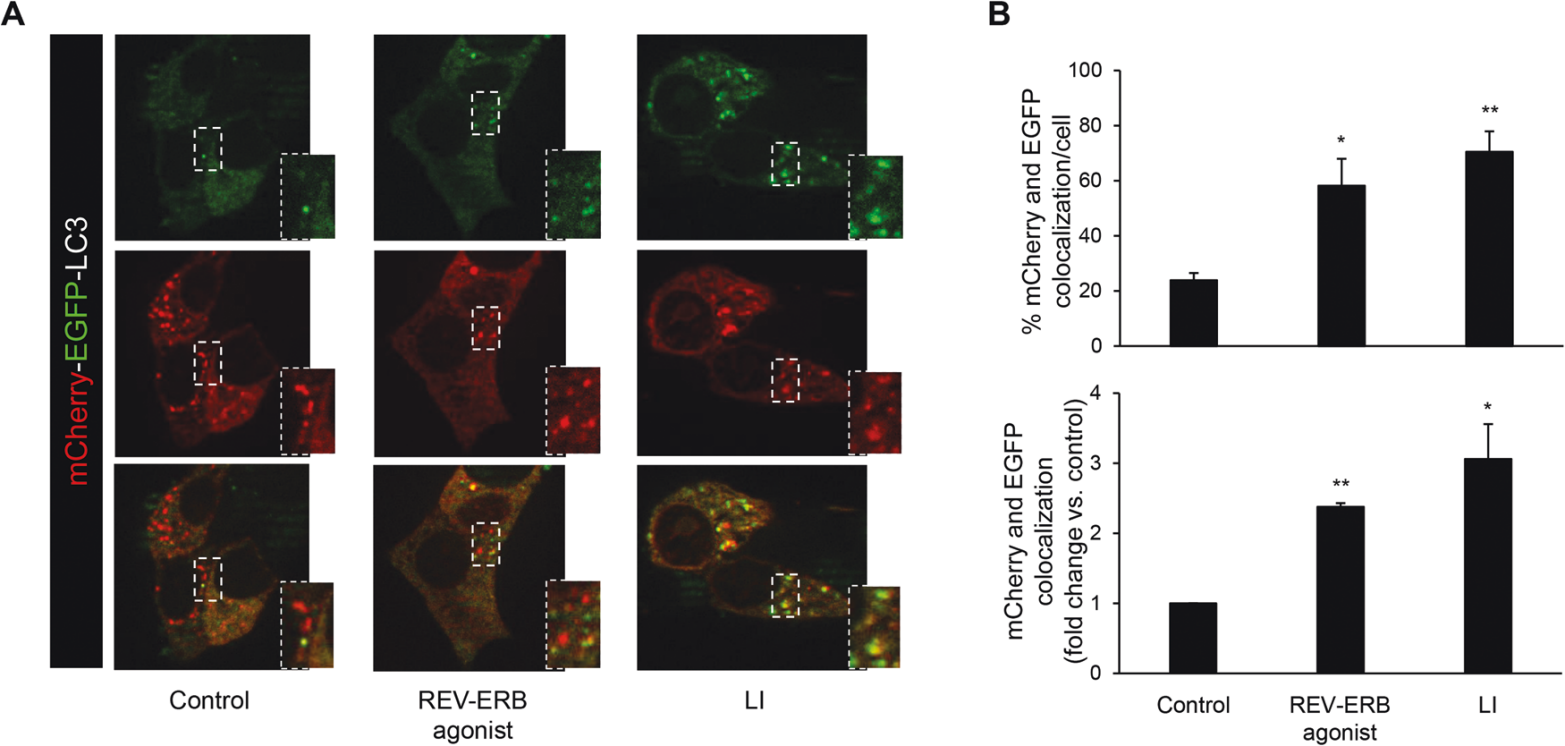
REV-ERBα agonism impairs autophagic flux in β-cells. **A** INS-1E cells expressing mCherry-EGFP-LC3B plasmid were either cultured in the absence or presence of lysosomal inhibitors (LI: E-64-d, 10 μg/ml and pepstatin A, 10 μg/ml, 24 h), or exposed to REV-ERB agonist (SR9009, 10 μM, 24 h) or control (DMSO). **B** Autophagic flux was assessed by quantification of mCherry and EGFP puncta colocalization using the Image J software. Results are expressed as the percentage of mCherry and EGFP colocalization per cell (top graph), and as fold change vs. control (bottom graph). Data are expressed as mean ± SEM of three to four independent experiments; *P < 0.05, **P < 0.01 vs. control.

To further expand on our observations, we next used a sensitive method to assess autophagic flux based on the expression of tandem-labeled mCherry-EGFP-LC3B [29]. This assay relies on different pH sensitivity of EGFP and mCherry fluorescent proteins: when the autophagosome fuses with the acidic environment of the lysosome, EGFP fluorescence is lost whereas mCherry fluorescence remains intact in acidic stores. Validation of this system in INS-1E cells confirmed that blockage of the autophagic flux with common lysosomal inhibitors (E-64-d and pepstatin A) or chloroquine promoted increased colocalization of EGFP and mCherry puncta (Supplemental Fig. 1A, B), as already reported for chloroquine [5]. Importantly, exposure of INS-1E cells to SR9009 increased colocalization of EGFP and mCherry puncta by 2.4 ± 0.05 fold (Fig. 3A, B, bottom graph, *P* < 0.01), further supporting an impaired autophagic flux under pharmacological activation of REV-ERBα.

**Fig. 4:**
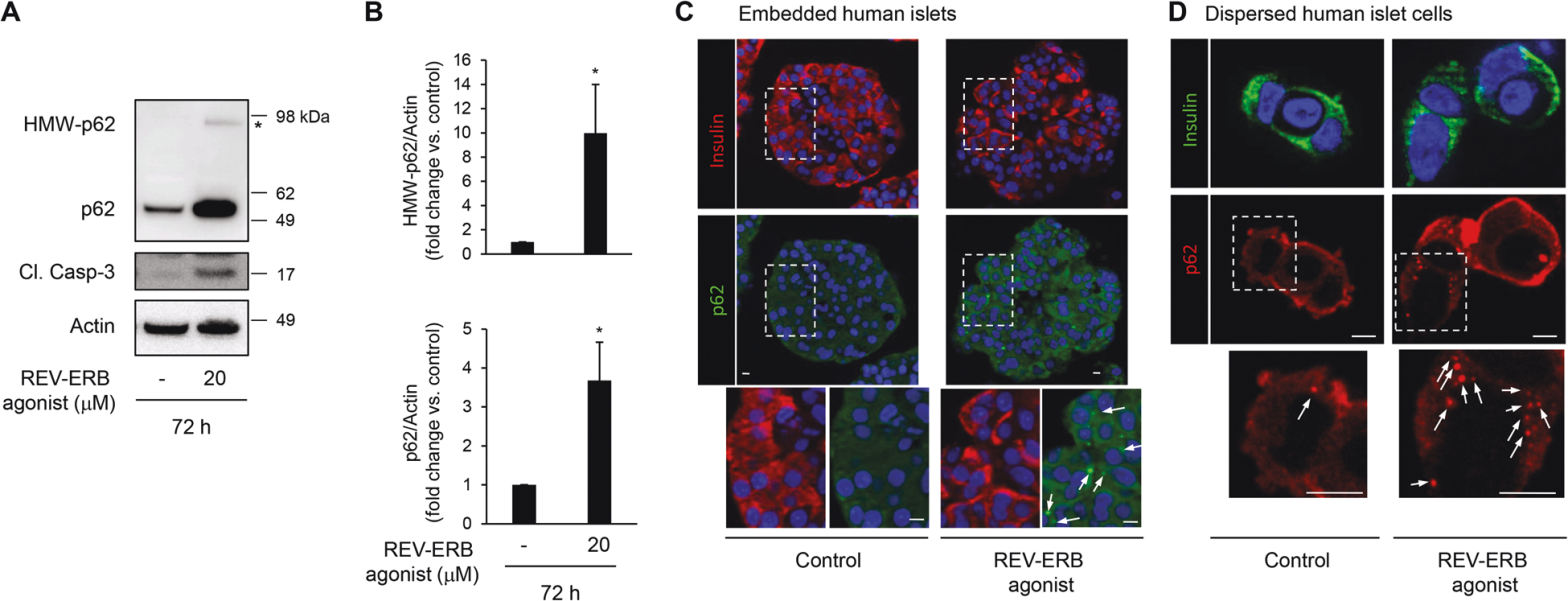
REV-ERBα agonism impairs autophagic flux in human islets. **A** Human islets were exposed to REV-ERB agonist (SR9009, 20 μM, 72 h) or control (DMSO). Protein levels of p62, high molecular weight (HMW)-p62, Cleaved Caspase-3, and actin (loading control) were analyzed by western blot. **B** Graphs represent the quantification of the blots (*n* = 4 independent experiments). Data are expressed as mean ± SEM; *P < 0.05 vs. control. **C** p62 staining was assessed by immunofluorescence (p62, green; insulin, red; nuclei, blue) in paraffin-embedded human islets exposed to REV-ERB agonist (SR9009, 20 μM, 72 h) or control (DMSO). **D** Dispersed human islets cells were exposed to REV-ERB agonist (SR9009, 20 μM, 24 h) or control (DMSO), and then fixed for immunofluorescence detection of p62 (red), insulin (green) and nuclei (blue). p62-positive cytoplasmic inclusions in β-cells are indicated with arrowheads. Scale bar: 5 μm.

To establish whether pharmacological activation of REV-ERBα inhibits autophagy in primary human β-cells, we evaluated p62 expression in isolated human islets treated with SR9009. Consistent with our previous observations, p62 protein levels were markedly increased (3.7 ± 0.9 fold, *P* < 0.05) in human islets treated with SR9009 and were associated with robust induction of Cleaved Caspase-3 (Fig. 4A). Similar to INS-1E cells, a p62 high molecular weight band (98 kDa) was detected under SR9009 exposure (Fig. 4A, B, *P* < 0.05). Furthermore, treatment of human islets with REV-ERB agonist resulted in detection by immunofluorescence of p62-positive cytoplasmic inclusions in human islet sections and in dispersed human β-cells (arrows in Fig. 4C, D), providing an additional hallmark of defective lysosomal degradation in β-cells [10, 28].

### Pharmacological activation of REV-ERBα attenuates β-cell secretory function

**Fig. 5:**
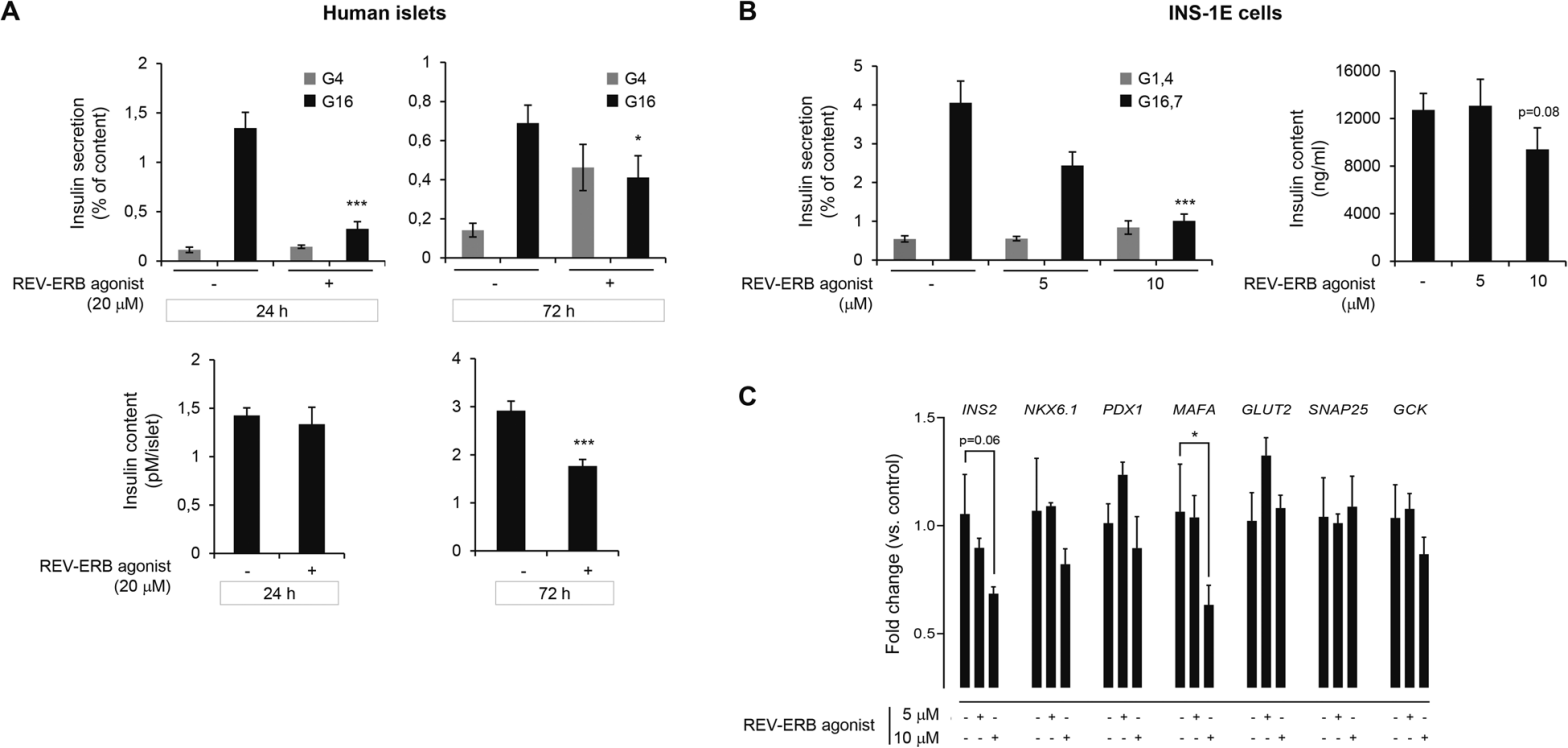
Pharmacological activation of REV-ERBα attenuates β-cell secretory function. **A** Isolated human islets were exposed to REV-ERB agonist (SR9009, 20 μM, for either 24 h or 72 h) or control (DMSO). Following a 30 min quiescent period in Krebs 4 mM glucose (G4), islets were stimulated with 16 mM glucose (G16) for 30 min at 37 °C. The graph represents insulin secretion normalized to insulin content (*n* = 7–8 independent experiments). The bottom panel represents insulin content in human islets. Data are expressed as mean ± SEM; *P < 0.05, ***P < 0.001 vs. control. **B** INS-1E cells were exposed to REV-ERB agonist (SR9009, 5 or 10 μM, 24 h) or control (DMSO). Following a 2 h quiescent period in Krebs 1.4 mM glucose (G1.4), cells were stimulated with 16.7 mM glucose (G16.7) for 1 h at 37 °C; (G1.4 refers to non-stimulated cells). The graph represents insulin secretion normalized to insulin content (*n* = 4 independent experiments). The bottom panel represents insulin content in INS-1E cells. **C** INS-1E cells were exposed to REV-ERB agonist (SR9009, 5 or 10 μM, 24 h) or control (DMSO). mRNA expression of key β**-**cell identity and functional genes expressed as fold change relative to control expression (*n* = 4 independent experiments per condition). Data are expressed as mean ± SEM; ***P < 0.001, *P < 0.05 vs. control.

Having established a potential repressive role of REV-ERBα on β-cell autophagy and survival, we next assessed its role in the regulation of glucose-stimulated insulin secretion. A short exposure of 24 h to REV-ERBα synthetic ligand SR9009 was sufficient to attenuate β-cell secretory function in isolated human islets as shown by the prominent decrease in 16 mM glucose-stimulated insulin secretion (Fig. 5A, top panel, *P* < 0.001 vs. vehicle in G16). This effect is unlikely to be caused by absolute insulin deficiency since SR9009 administration failed to alter overall insulin content (Fig. 5A, bottom panel). In contrast, prolonged exposure to SR9009 (72 h) resulted in the overall decline in islet insulin content, the elevation of basal insulin release, and a decrease in glucose-stimulated insulin secretion (Fig. 5A, *P* < 0.05 vs. vehicle in G16). Similarly, a 24-h exposure of INS-1E cells to SR9009 (10 μM) resulted in a reduced insulin secretory function (Fig. 5B, *P* < 0.001 vs. vehicle in G16.7). As shown by corresponding gene expression analysis in INS-1E cells, the SR9009-mediated decrease in β-cell function was not associated with attenuated expression of genes regulating glucose sensing and exocytosis (e.g., *GLUT2*, *SNAP25*, *GCK*), but rather appeared to alter the expression of β-cell identity genes (e.g., *INS2* and *MAFA*) (Fig. 5C). Overall, these results indicate that pharmacological activation of REV-ERBα also compromises β-cell function, an observation consistent with the previously proposed role of autophagy in the regulation of β-cell survival, insulin biosynthesis, and β-cell homeostasis [30, 31].

### Negative modulation of REV-ERBα protects β-cells and human islets from inflammation and glucotoxicity-induced apoptosis

**Fig. 6:**
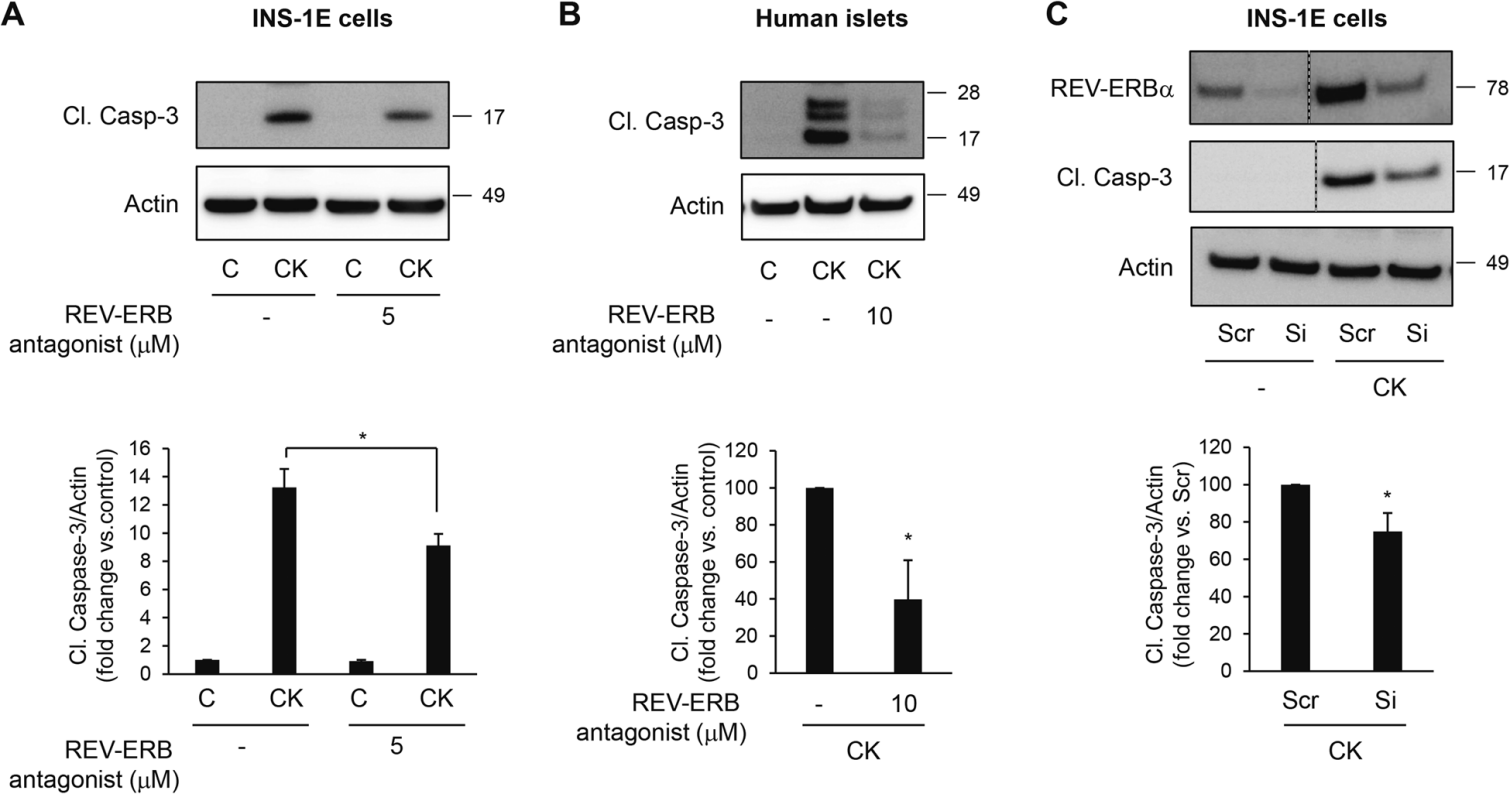
Negative modulation of REV-ERBα protects β-cells and human islets from cytokines-induced apoptosis. INS-1E cells (**A**) or human islets (**B**) were exposed or not to pro-inflammatory cytokine mix (CK) in the presence or absence of REV-ERB antagonist (SR8279, 5 or 10 μM for 24 h). Cleaved Caspase-3 and actin (loading control) levels were analyzed by western blot (*n* = 3–4 independent experiments). **C** REV-ERBα was silenced in INS-1E cells by siRNA. Scramble RNA (Scr) was used as a control. Cells were then exposed to pro-inflammatory cytokine mix (CK). Levels of REV-ERBα, Cl. Caspase-3, and actin were analyzed by western blot (*n* = 3 independent experiments). For REV-ERBα and Cl. Caspase-3 images, lanes were run on the same gel but were non-continuous (as indicated). Data are expressed as mean ± SEM; *P < 0.05 vs. control.

**Fig. 7:**
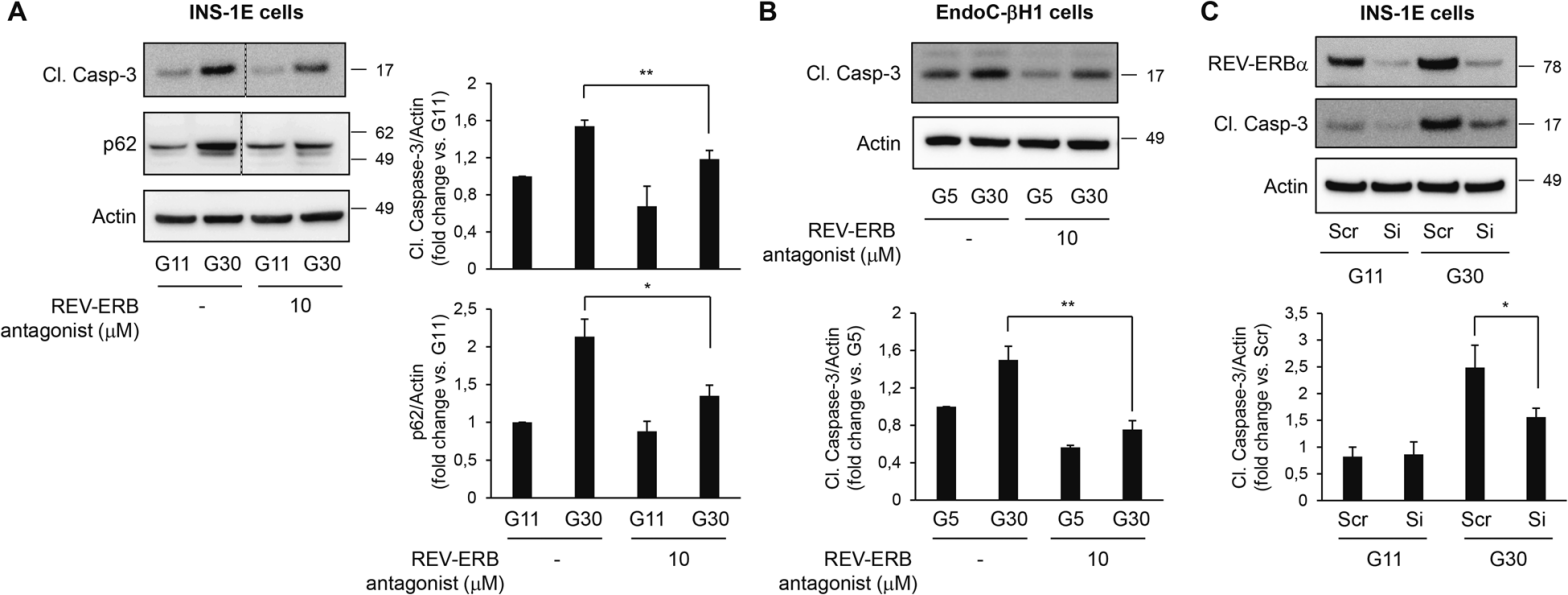
Negative modulation of REV-ERBα protects β-cells from glucotoxicity-induced apoptosis. INS-1E (**A**) or EndoC-βH1 cells (**B**) were exposed to glucotoxicity [30 mM glucose (G30) vs. control 11 mM glucose (G11)] for 48 h in the presence or absence of REV-ERB antagonist (SR8279, 10 μM). Cleaved Caspase-3, p62, and actin levels (loading control) were analyzed by western blot (*n* = 5 independent experiments for INS-1E; *n* = 3 independent experiments for EndoC-βH1). **C** REV-ERBα was silenced in INS-1E cells by siRNA. Scramble RNA (Scr) was used as a control. Cells were then exposed to glucotoxicity for 48 h (G30 vs. G11). Levels of REV-ERBα, Cl. Caspase-3, and actin were analyzed by western blot (*n* = 4 independent experiments). For p62 and Cl. Caspase-3 images, lanes were run on the same gel but were non-continuous (as indicated). Data are expressed as mean ± SEM; *P < 0.05, **P < 0.01 vs. control.

Given the purported involvement of REV-ERBα in the regulation of β-cell dysfunction and apoptosis, we next evaluated whether the negative modulation of REV-ERBα could protect β-cells from pro-diabetogenic stressors. We first explored whether inhibition of REV-ERBα activity using the chemical antagonist SR8278 [32] attenuates cytokine-mediated β-cell apoptosis (Fig. 6). Our experiments revealed a ~30% decrease in cytokines-induced INS-1E cell apoptosis (Fig. 6A, *P* < 0.05) and a ~60% decrease in cytokine-induced human islet cell apoptosis in the presence of REV-ERBα antagonist (Fig. 6B, *P* < 0.05). To further confirm these findings, we next used a siRNA approach to specifically target *Rev-erbα* expression. We validate our approach by demonstrating that transfection of INS-1E cells with 10 nM of siRNA for 48 h resulted in a ~75% knockdown of REV-ERBα protein expression under both control and cytokine-stimulated conditions (Fig. 6C). Importantly, siRNA-mediated decrease in REV-ERBα resulted in significant protection of INS-1E cells from cytokines-induced apoptosis (25% decrease, Fig. 6C, *P* < 0.05). Under glucotoxicity, pharmacological inhibition of REV-ERBα decreased apoptosis by 23% in INS-1E cells (Fig. 7A, *P* < 0.01) and by 50% in human EndoC-βH1 cells (Fig. 7B, *P* < 0.01). Consistently, specific knockdown of *Rev-erbα* by siRNA (10 nM) resulted in a 37% decrease in glucotoxicity-induced apoptosis (Fig. 7C, *P* < 0.05).

**Fig. 8:**
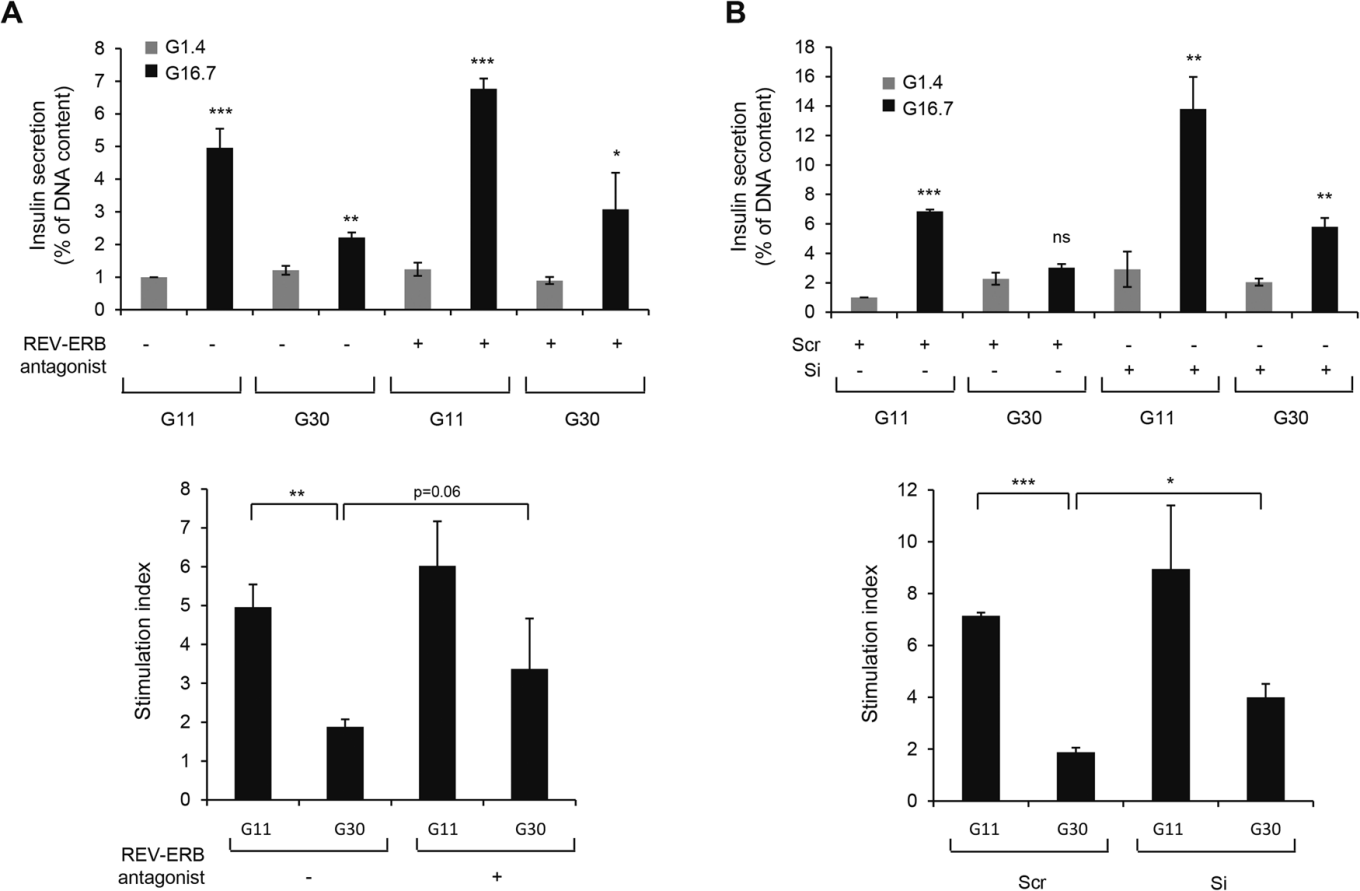
Negative modulation of REV-ERBα partially protects from glucotoxicity-induced β-cell dysfunction. **A** INS-1E cells were exposed to glucotoxicity [30 mM glucose (G30) vs. control 11 mM glucose (G11)] for 48 h in the presence or absence of REV-ERB antagonist (SR8279, 10 μM). Following a 2 h quiescent period in Krebs 1.4 mM glucose (G1.4), cells were stimulated with 16.7 mM glucose (G16.7) for 1 h at 37 °C; (G1.4 refers to non-stimulated cells). The graph represents insulin secretion normalized to DNA concentration (*n* = 4 independent experiments). **B** REV-ERBα was silenced in INS-1E cells by siRNA. Scramble RNA (Scr) was used as a control. Cells were then exposed to glucotoxicity for 48 h (G30 vs. G11). Following a 2 h quiescent period in Krebs 1.4 mM glucose (G1.4), cells were stimulated with 16.7 mM glucose (G16.7) for 1 h at 37 °C; (G1.4 refers to non-stimulated cells). The graph represents insulin secretion normalized to DNA concentration (*n* = 4 independent experiments). The bottom graphs represent the stimulation index (ratio G16.7: G1.4). Data are expressed as mean ± SEM; *P < 0.05, **P < 0.01, ***P < 0.001 vs. G1.4 (unless specified).

In addition, impaired glucose-stimulated insulin secretion under glucotoxicity had the tendency to be improved by pharmacological inhibition (Fig. 8A, bottom panel, *P* = 0.06 vs. vehicle in G30) and was significantly ameliorated by specific knockdown of *Rev-erbα* (Fig. 8B, bottom panel, *P* < 0.05 vs. Scr in G30) in INS-1E cells. This partial improvement of β-cell function under negative modulation of REV-ERBα was unlikely to be caused by changes in β-cell function/identity gene expression (Supplemental Fig. 2A, B). Notably, the antagonist SR8279 decreased glucotoxicity-induced p62 accumulation (Fig. 7A, *P* < 0.05), thus suggesting that inhibition of REV-ERBα attenuated β-cell apoptosis and dysfunction by restoring impaired autophagic flux.

### Digital genomic footprinting reveals that REV-ERBα preferentially binds to genomic loci regulating protein degradation and autophagy in T2DM human islets

**Fig. 9:**
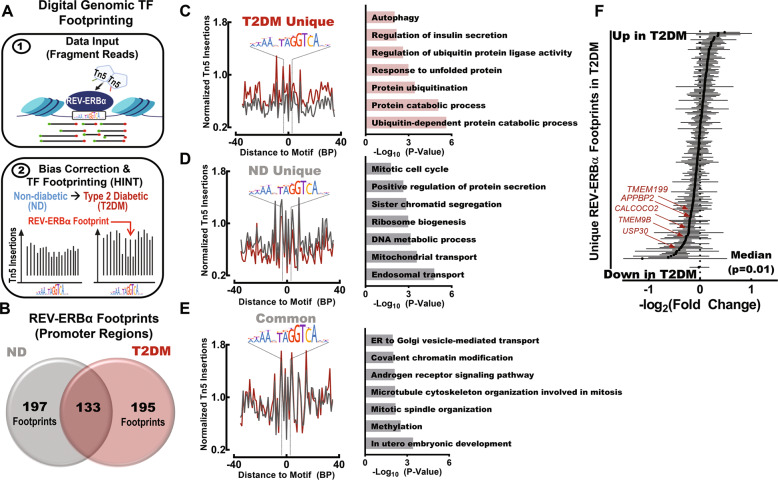
REV-ERBα preferentially binds to promoter regions of genes regulating protein degradation and autophagy in T2DM human islets. **A** Schematic outlining digital genomic transcription factor (TF) footprinting from ATAC-seq of ND and T2DM islet samples (SRP117935) [35]. Transcription factors (e.g., REV-ERBα) protect against Tn5 digestion, resulting in a local decrease in chromatin accessibility (‘footprint’). This enables for identification and differential analysis of transcription factor binding at regulatory motifs in non-diabetic (ND) versus Type 2 Diabetic (T2DM) islets using the Hmm-based IdeNtification of Transcription factor footprint framework (HINT) [36]. **B** Venn diagram highlighting the overlap of REV-ERBα footprints identified in promoter regions (±1 kb from TSS) from at least two ND and/or T2DM islet samples (*n* = 5 ND and *n* = 5 T2DM samples). **C**–**E** (left): Representative ND and T2DM REV-ERBα footprints uniquely observed in T2DM islets (top), uniquely in ND islets (middle), and common (bottom) in both ND and T2DM islets within promoter regions. The signal is normalized to the total read depth of each sample and is plotted ±35 bp from the REV-ERBα motif center. **C**–**E** (right): Enriched GO: Biological Process pathways annotated from T2DM unique (top), ND unique (middle), and common (bottom) REV-ERBα footprints in promoter regions. **F** Forrest plot illustrating results of the meta-analysis for genes annotated as REV-ERBα footprints (unique to T2DM) derived from 5 publicly available human islet microarray datasets comparing the transcriptome of non-diabetic and type 2 diabetic islets (GSE38642 [63], GSE 25724 [64], GSE20966 [65], GSE76894 [66], and GSE76895 [66]). Red arrows highlight common genetic regulators of autophagy and protein degradation in T2DM.

Computational approaches have been recently developed to analyze transcription factor binding and activity at a genome-wide level taking advantage of the fact that bound transcription factors impede DNA cleavage thus creating areas of lower Tn5 transposase insertion in larger accessible chromatin regions (e.g., “footprints”) (Fig. 9A) [33, 34]. Thus, to extend our findings to T2DM conditions, we assessed publicly available assay for transposase-accessible chromatin using sequencing (ATAC-seq) libraries and corresponding peak sets from 5 non-diabetic (ND) and 5 T2DM pancreatic islet donors (SRP117935) [35]. In order to define genome-wide REV-ERBα binding to *cis*-regulatory elements in ND and T2DM islets, we utilized Hmm-based IdeNtification of Transcription factor footprints (HINT) [36]. Using this method, we observed robust differences in REV-ERBα activity between non-diabetic vs T2DM islets (Fig. 9B). Most notably, REV-ERBα footprints unique for T2DM islets were annotated to promoter regions representing genomic loci associated with protein degradation and autophagy pathways such as GO: ~protein catabolic process, ~protein ubiquitination, and ~autophagy (Fig. 9C). Interestingly, non-diabetic conditions were associated with unique REV-ERBα footprints in genomic regions associated with GO: ~endosomal transport, ~mitochondrial transport, and ~mitotic cycle (Fig. 9D), whereas REV-ERBα footprints common to non-diabetic and T2DM conditions annotated to genomic loci associated with GO: ~methylation, ~cytoskeleton organization and covalent chromatin organization (Fig. 9E). Finally, a meta-analysis of publicly available 5 human islet microarray datasets (GSE25724, 76894, 38642, 76895, and 20966) comparing the transcriptome of ND and T2DM islets revealed significant downregulation of genes annotated as REV-ERBα footprints (unique to T2DM conditions) in human T2DM islets (Fig. 9F, *P* = 0.01). As annotated in Fig. 9F, this analysis notably reveals the downregulation of genes involved in autophagy induction (*CALCOCO2*), lysosomal acidification (*TMEM199, TMEM9B*), mitophagy (USP30), and microtubule dynamics (*APPBP2*) required for autophagosome formation and motility [37].

## Discussion

The etiology of β-cell failure in T2DM is driven by a complex interaction between genetic susceptibilities and exposure to pro-diabetogenic stressors typically attributed to the induction of obesity and insulin resistance [38]. Most prominent diabetogenic stressors include chronic exposure to elevated glucose concentrations and pro-inflammatory cytokines which converge to promote induction of oxidative/endoplasmic reticulum (ER) stress and significantly compromise β-cell secretory function and survival [39, 40]. At the molecular level, β-cells in T2DM are characterized by disrupted regulation of autophagy, which is particularly damaging to β-cells given the characteristic high burden of protein synthesis and folding in obesity and T2DM. Subsequently, our study provides the first evidence that glucotoxicity and inflammation-mediated disruption of autophagy and consequent induction of β-cell apoptosis is driven in part by augmented expression and activity of REV-ERBα. Importantly, our study also found that negative modulation of REV-ERBα expression provides protection from glucotoxicity and inflammation-mediated β-cell failure. Finally, utilizing novel bioinformatics approaches we also provide evidence that augmented REV-ERBα activity in T2DM human islets is associated with impaired transcriptional regulation of autophagy, protein degradation, and ubiquitination pathways. Given the key role of the ubiquitination process in autophagy [27] and the existence of interplay between autophagy and the ubiquitin-proteasome system in β-cells [41], it is not surprising that these data further reveal a wider role of REV-ERBα in the modulation of protein degradation in T2DM islets.

Our results obtained in both clonal and human β-cells demonstrate that pharmacological activation of REV-ERBα with the synthetic ligand SR9009 disrupts autophagy; however, the mechanisms underlying this observation are not fully understood. By the demonstration of p62 accumulation (cytosolic inclusions and protein levels) and the use of the tandem mCherry-EGFP-LC3 reporter system, our data revealed a REV-ERBα-mediated blockade in lysosomal degradation, therefore, impacting the autophagic flux. Since autophagosome number was rather increased in β-cells exposed to REV-ERBα agonist, lysosomal clearance seems most likely affected but we cannot exclude a simultaneous role of REV-ERBα on autophagic vacuole formation and lysosomal degradation in β-cells. Indeed, recent studies in skeletal muscle and cancer cells reported that pharmacological activation of REV-ERBα inhibits autophagosome formation via negative modulation of core autophagy genes such as *Ulk3*, *Ulk1*, *Bec1*, *Atg7*, and *Atg5* [17, 18]. Similarly, REV-ERBα-mediated repression of autophagy was recently shown in zebrafish where investigators utilized ChIP assays to demonstrate the direct binding of REV-ERBα to promoter regions of key autophagy genes (e.g., *ulk1a* and *atpv1d*). Consistently, REV-ERBα deficient zebrafish were characterized by significant augmentation in the number of autophagosomes/autolysosomes and the expression of autophagy-related genes [42].

It is becoming increasingly evident that autophagy is a critical component of β-cell homeostasis in health and under conditions associated with diabetogenic stress [43]. Specifically, autophagy is required for proper control of β-cell development and maturation and plays an important role in the regulation of insulin biosynthesis and secretion [30, 44]. Importantly, autophagy is also essential for β-cell survival in response to conditions associated with ER stress and oxidative stress as commonly occurs in β-cells exposed to glucotoxicity and inflammation in T2DM [31, 45]. Indeed, inhibition of autophagy compromises β-cell survival and leads to dysregulation of glucose homeostasis in response to diet-induced obesity and/or increased expression of human islet amyloid polypeptide (hIAPP) [9, 46]. In contrast, stimulation of autophagy via pharmacological strategy (e.g., rapamycin) has been shown to preserve β-cell integrity under diabetogenic conditions [31, 45, 47]. Our data is consistent with these observations and demonstrates for the first time that REV-ERBα antagonist or knock-down provides protection from glucotoxicity and cytokine-mediated β-cell apoptosis through stimulation of autophagy. In addition, given the closely linked relationship between β-cell secretory function and mass [48], negative modulation of REV-ERBα may also improve β-cell function under glucotoxicity through stimulation of autophagy and decreased apoptosis. However, future studies will be required to assess the potency of REV-ERBα inhibition as a strategy to promote β-cell survival and function in diabetes.

Consistent with recent observations [19, 20], our data reveal an upregulation of REV-ERBα in β-cells exposed to diabetes-related conditions. REV-ERBα is a transcriptional repressor that has been shown to negatively regulate the expression of genes involved in many important cellular processes such as metabolism, inflammation, proliferation, and notably, plays an important role in the stabilization of the core circadian clock transcriptional-translational feedback look. Indeed, both *BMAL1* and *CLOCK* are direct target genes of REV-ERBα, thus chronic pharmacological activation of REV-ERBα (e.g., with synthetic agonist SR9009) suppresses *BMAL1* expression and imparts disruptive effects on the circadian clock [26]. This suggests that glucotoxicity and inflammation-mediated increase in β-cell REV-ERBα expression may also promote β-cell failure indirectly through suppressive effects of REV-ERBα on the core circadian clock. Conversely, negative modulation of REV-ERBα may also improve β-cell function under glucotoxicity via upregulation of *BMAL1*. This is in line with a recent observation that clock amplitude-enhancing compound Nobiletin (as opposed to SR9009) enhances in vitro glucose-stimulated insulin secretion in T2DM human islets, whereas enhancement of β-cell circadian clock in vivo via conditional overexpression of *Bmal1* improves insulin secretion and glucose tolerance in mice exposed to diet-induced obesity [49, 50]. Whereas these effects are likely mediated through modulation of circadian amplitude in islets, as shown for Nobiletin and *Bmal1* β-cell overexpression [49, 50], we speculate that deleterious effects of REV-ERBα activation on β-cell autophagy and survival are likely attributed to mechanisms independent of core circadian clock transcription factors (e.g., BMAL1 and CLOCK). Indeed, although BMAL1 and CLOCK target genes are enriched for regulators of glucose-stimulated insulin secretion and insulin exocytosis, these transcription factors do not appear to be involved in the transcriptional control of autophagy and cell survival [51]. In addition, whereas REV-ERBα agonism did not repress the expression of genes regulating β-cell insulin secretory mechanism, our studies cannot rule out the possibility that REV-ERBα activation may also alter β-cell secretory function and survival through a transcriptional repressor activity (independently of autophagy).

Using the rat pancreatic β-cell line INS-1E and human β-cell line/islets, we reported that activation of REV-ERBα impaired insulin secretion and inhibition protected β-cells from diabetogenic conditions. These data are in contradiction with the study from Vieira et al. [52] reporting that REV-ERBα silencing impaired glucose-stimulated insulin secretion in the mouse pancreatic β-cell line (MIN6) and in mouse islets. The contradiction of these results can rely on two main points: 1/ the differences between the β-cell lines that were used: MIN6 vs INS-1E cells. Supporting this assumption, opposite results between MIN6 and INS-1 cells have already raised a concern regarding the role of REV-ERBα in the regulation of lipogenic genes expression [52, 53]. These discrepancies underlie the importance to validate key results in human pancreatic β-cells to avoid species-specific effects. The data we obtained in INS-1E cells are reproducible in human islets and human β-cells, reinforcing our confidence in the proposed conclusions; 2/ the context of negative modulation of REV-ERBα: in contrast to Vieira et al. [52] who investigated the consequences of REV-ERBα invalidation in healthy β-cells, we knocked down REV-ERBα in the context of severe β-cell failure (glucotoxic conditions) in order to evaluate any beneficial effects on β-cell function.

To examine the pathological role of REV-ERBα activation in β-cells, we investigated the effects of synthetic ligand SR9009 which has been shown to directly bind REV-ERBα and enhance its repressor activity [26]. Indeed, our study established that SR9009-mediated activation of REV-ERBα disrupts β-cell autophagy, insulin secretory function, and survival, reproducing many aspects of the pathophysiology of human islets in T2DM. However, it is important to note that potential off-target effects of SR9009 were recently described in hepatocytes thus raising concerns regarding REV-ERBα-specific actions of SR9009 [54]. Although the specificity of this compound for REV-ERBα activation is supported by loss-of-function studies in REV-ERBα-deficient mice [23, 25], future work will be important in confirming the specificity of REV-ERBα activation in the modulation of β-cell failure in T2DM.

In conclusion, our study demonstrates a previously unexplored link between the core circadian clock nuclear receptor REV-ERBα, autophagy, and β-cell failure in response to diabetogenic conditions. Our observation that the negative modulation of REV-ERBα (either by pharmacological or specific genetic inhibition) provides partial protection for glucotoxicity- and cytokines-induced β-cell apoptosis point to REV-ERBα as a potential new therapeutic target to modulate autophagy, protein degradation and β-cell survival in diabetes. These observations are in line with recent studies demonstrating an important role of REV-ERBα in the regulation of inflammation and amyloid clearance in Alzheimer’s disease [55, 56]. Future studies are warranted to explore mechanisms underlying increased REV-ERBα expression and transcriptional activity in human T2DM β-cell and the corresponding potential of REV-ERBα-based strategies to preserve a functional β-cell mass in T2DM.

## Materials and methods

### Human islets/dispersed human islet cells

Experiments involving human islets were performed in agreement with the local ethic committee (Biological Ressources Center, Collection IRB 5 “Human Islets of Langerhans”, identifiant Biobanque no. BB-0033-00031, CHU, Montpellier), the institutional ethical committee of the French Agence de la Biomédecine (ABM no. PFS 13-008) and the French Ministry of Research (DC-2011-1401 and AC-2017-3039). Informed consent was obtained for all pancreases. Pancreases were harvested from five brain-dead non-diabetic donors. Isolated islets were prepared by collagenase digestion followed by density gradient purification at the Laboratory of Cell Therapy for Diabetes (Institute for Regenerative Medicine and Biotherapy, Montpellier, France), according to a slightly modified version of the automated method [57]. Following isolation, human islets were cultured for recovery for 3 days at 37 °C, in a 5% CO_2_ atmosphere, in CMRL 1066 medium (Life Technologies) containing 5.6 mM glucose supplemented with 10% FBS, 2 mM glutamine, 25 mM HEPES, 100 IU/ml penicillin, and 100 μg/ml streptomycin. Islets were then incubated in CMRL 1066 medium containing 5.6 mM glucose. The islet purity was 90%, as re-assessed by dithizone staining in the host laboratory. REV-ERB agonist (SR9009, resuspended in DMSO; Millipore) was added at 20 μM for 72 h in CMRL 1066 medium. For pro-inflammatory cytokine exposure, human islets were incubated in a cytokine mix [100 U/ml IL-1β (2 ng/ml), 500 U/ml TNF-α (50 ng/ml) and 100 U/ml IFN-γ (33 ng/ml)] for 24 h. Murine recombinant IFN-γ was from Life Technologies; murine IL-1β and TNF-α from PeproTech. REV-ERB antagonist (SR8278, resuspended in DMSO; Millipore) was added at 10 μM for 24 h. At the end of the experiment, islets were washed with cold PBS and lysed for 10 min at 4 °C in NP40 lysis buffer [58], sonicated for 10 s, and centrifuged at 10,000 r.p.m. for 10 min. Clusters of cells were prepared by dispersion of isolated islets with trypsin and plated on glass coverslips in CMRL 1066 medium. REV-ERB agonist (SR9009) was added at 20 μM for 24 h.

### Cell culture

#### INS-1E cells

The rat β-cell line INS-1E was provided by Dr. P. Maechler (Department of Cell Physiology and Metabolism, University of Geneva, Geneva, Switzerland) [59]. INS-1E cells were grown in RPMI-1640 medium with 11 mM glucose supplemented with 7.5% heat-inactivated FBS, 1 mM sodium pyruvate, 50 *μ*M *β*-mercaptoethanol, 2 mM glutamine, 10 mM HEPES, and 100 IU/ml penicillin and 100 *μ*g/ml streptomycin (Life Technologies) at 37 °C in a humidified 5% CO_2_ atmosphere. No mycoplasma contamination was detected. Soluble and insoluble fractions were prepared using the ReadyPrep Protein Extraction Kit (Soluble/Insoluble) (Bio-Rad, 163-2085) according to the manufacturer’s instructions. For glucotoxicity experiments, INS-1E cells were cultured in a complete RPMI 1640 medium (Life Technologies) containing 11- or 30-mM glucose for 48 h. For pro-inflammatory cytokine exposure, INS-1E cells were incubated in a cytokine mix [10 U/ml IL-1*β* (0.2 ng/ml), 500 U/ml TNF-*α* (50 ng/ml) and 100 U/ml IFN-*γ* (33 ng/ml)] for 24 h. REV-ERB agonist (SR9009, resuspended in DMSO; Millipore) was added at 5 or 10 µM for 24 h. REV-ERB antagonist (SR8278, resuspended in DMSO; Millipore) was added at 5 or 10 µM during the last 24 h of treatment.

#### EndoC-βH1 cells

The human β-cell line EndoC-βH1 was provided by Univercell-Biosolutions (Toulouse, France) and cultured as previously described [60]. Briefly, cells were seeded at a density of approximately 600,000 cells/cm^2^ on tissue culture-treated plates pre-coated overnight with coating matrix in EndoC-βH1 complete medium (Univercell-Biosolutions). Cells were passaged approximately every 7 days. All experiments on EndoC-βH1 were performed from passage 10 to 45 after thawing. For glucotoxicity experiments, EndoC-βH1 cells were cultured in a complete medium containing 5.6- or 30-mM glucose for 72 h. REV-ERB antagonist (SR8278, resuspended in DMSO; Millipore) was added at 10 μM during the last 24 h of treatment. At the end of the experiment, cells were washed with cold PBS and lysed for 10 min at 4 °C in NP40 lysis buffer and centrifuged at 10,000 r.p.m. for 10 min.

### REV-ERBα siRNA experiments

REV-ERBα expression was silenced in INS-1E cells using Silencer Select siRNA duplexes designed for rat *Nr1d1* (s141182, Life Technologies). Cells were seeded in 6-well plates at 800,000 cells/well and grown overnight to reach 40–50% confluency. The next day, lipofectAMINE3000-siRNA complexes were prepared according to the manufacturer’s instructions. REV-ERBα siRNA duplexes were tested at final concentrations of 10, 25 or 50 nM. Cells were transfected with REV-ERBα siRNA or control siRNA (scramble) (10 nM final concentration) in Opti-MEM (Life Technologies) for 24 h before switching to the fresh culture medium, glucotoxicity condition (48 h) or cytokine mix (24 h).

### Western blotting

Proteins (25–50 μg/lane) were separated on a 4–12% Bis-Tris NuPAGE gel and blotted onto a PVDF membrane (FluoroTrans; VWR). Membranes were probed overnight at 4 °C with primary antibodies against REV-ERBα (13418, Cell signaling), LC3B (2775, Cell signaling), cleaved caspase-3 (9661, Cell signaling), p62 (GP62-C, Progen, Heidelberg, Germany), Actin (A5441, Sigma). Horseradish peroxidase-conjugated secondary antibodies were from Cell Signaling. Proteins were visualized by enhanced chemiluminescence (Millipore) on ChemiDoc camera (Bio-Rad) and protein expression levels were quantified using the ImageJ software (National Institutes of Health, Bethesda, MD). Full and uncropped western blots are presented in Supplemental File.

### RNA isolation and quantitative, real-time PCR analysis (qRT-PCR)

Total RNA was extracted using the RNeasy Mini Kit (Qiagen, Courtaboeuf, France) according to the manufacturer’s instructions. In all, 300 ng of total RNA was subsequently transcribed into cDNA using the iScript cDNA Synthesis Kit (Bio-Rad, 1708891). Gene-specific primers (Supplemental Table 1) were mixed with the cDNA and SYBR Green Master Mix (Applied Biosystems, Waltham, MA), and analysis was performed by the Applied Biosystems StepOnePlus Real-Time PCR System. *β-actin* expression was used for normalization of results.

### Immunostaining and imaging

Isolated human islets were treated with REV-ERB agonist (SR9009, 20 μM, 72 h) or DMSO as the vehicle (130 islets per condition). Islets were then washed twice in PBS and transferred into 3.7% paraformaldehyde (Sigma-Aldrich) in PBS for fixation for 4 h. After 2 washes in PBS, islets were colored in 0.1% neutral red, washed twice in PBS, and embedded in 24% agar. Cubes of agar containing batches of islets were fixed overnight into 3.7% paraformaldehyde, washed with PBS before being transferred into 70% ethanol, and then processed for paraffin embedding. Sections were then incubated overnight at 4 °C with primary antibodies (anti-p62: GP62-C, 1/100, Progen; anti-insulin, 1/100, Cell Signaling), followed by 1 h at room temperature with secondary antibodies and DAPI. Sections were mounted in Mowiol (Sigma-Aldrich). Fixed dispersed human islet cells were incubated overnight at 4 °C with primary antibodies (anti-p62: GP62-C, 1/400, Progen; anti-insulin, 1/200, Cell Signaling), followed by 1 h at room temperature with secondary antibodies and DAPI. For mCherry-EGFP-LC3B plasmid transfection experiments (pBABE-puro mCherry-EGFP-LC3B was a gift from Jayanta Debnath, Addgene #22418, Teddington, UK [61]), INS-1E cells were plated on 6-well plates at a density of 1 million cells/well. The next day, lipofectAMINE2000-mCherry-EGFP-LC3B plasmid complexes were prepared according to the manufacturer’s instructions. Cells were transfected in Opti-MEM (Invitrogen) for 4 h and then cultured in a complete medium for the indicated time. Images were acquired with an AxioImager Apotome microscope (Zeiss) using either a 40X (islets) or 100X (clusters of human cells and INS-1E cells) immersion oil objective and the Zen Blue software. For mCherry-EGFP-LC3B plasmid quantification, cells expressing mCherry were selected randomly and acquired to be blinded for EGFP expression. Ten fields per coverslip were imaged (as n = 1) and saved in “czi” (rather than “tiff”) format allowing to save the entire image parameters; they were reopened and quantified using ImageJ. Dots were counted manually offline on images whose identification was blinded to the experimenter.

### Measurements of insulin secretion

INS-1E cells were pre-incubated for 2 h in Krebs Ringer buffer (KRB) [62] containing 1.4 mM glucose, followed by a 1 h incubation at 1.4 or 16.7 mM glucose. Supernatant from the incubation buffers was collected and cleared by centrifugation. Insulin content extraction was performed using acid ethanol. Insulin release and contents were measured by homogenous time-resolved fluorescence (HTRF) (Cisbio bioassays, Codolet, France) according to the manufacturer’s instructions. HTRF signals were measured using Pherastar FS (BMG Labtech, Ortenberg, Germany) microplate reader. Insulin release was normalized to insulin or DNA content (as indicated). To measure static glucose-stimulated insulin secretion, human islets were incubated first in KRB buffer supplemented with 4 mM glucose for 30 min followed by exposure to KRB buffer containing 16 mM glucose for 30 min. Insulin was measured by using a human insulin ELISA kit (ALPCO). Insulin values were normalized to insulin content.

### Bioinformatics assessment of REV-ERBα genomic footprinting in human islets

Publicly available Assay for Transposase-Accessible Chromatin using sequencing (ATAC-seq) libraries and corresponding peak-sets from 5 non-diabetic (ND) and 5 type 2 diabetic pancreatic islet donors with comparable demographics were obtained from SRP117935 [35]. In order to define genome-wide REV-ERBα binding to cis-regulatory elements in ND and T2DM islets, we utilized Hmm-based IdeNtification of Transcription factor footprints (HINT) [36]. Raw binary alignment maps (BAM) and browser extensible data peak files (BED) were first imported into HINT and subsequently utilized to identify REB-ERBα binding sites using the function *rgt-hint footprinting* with the following options: *--atac-seq, --paired-end, --organism* = *hg19*. The HOmo sapiens COmprehensive MOdel Collection (HOCOMOCO) version 11 transcription factor binding model was used to define REV-ERBα binding sites. REV-ERBα footprints observed in ≥2 ND or T2DM samples were retained for downstream analysis. To examine REV-ERBα binding in promoter regions (±1 kb from TSS), we annotated REV-ERBα footprints using the Genomic Regions Enrichment of Annotations Tool (GREAT) v4.0.4 and subsequently used BEDTools v2.29.2 to calculate common and differential REV-ERBα binding within these regions. Enrichment of annotated REV-ERBα footprints to GO: Biological Process and Molecular Function pathways were performed by GREAT.

### Meta-analysis of publicly available human islet transcriptomes of non-diabetic (ND) and type 2 diabetic (T2DM) islets

Publicly available human islet microarray datasets comparing the transcriptome of non-diabetic and type 2 diabetic islets were queried using the National Center for Biotechnology Information’s Gene Expression Omnibus (GEO) using the search terms: “T2D/type 2 diabetes,” “pancreatic islet/islet,” and “Homo sapiens.” From our search, we obtained 5 studies containing at least five non-diabetic and five type 2 diabetic donors: GSE38642 [63], GSE 25724 [64], GSE20966 [65], GSE76894 [66], and GSE76895 [66]. The raw data was downloaded from https://www.ncbi.nlm.nih.gov/geo/query/acc.cgi?acc=GSEXXXXX. In studies where glycemia was assessed (GSE38642; GSE78695), non-diabetic samples with impaired glucose tolerance (HbA1c > 5.7% or noted), unknown glycemia, and type 3 diabetes were excluded. A total of 156 non-diabetic and 81 type 2 diabetic samples were retained for downstream analysis. The raw.CEL files were loaded into the R package Affy v1.68 for normalization of probe intensity by the MAS 5.0 algorithm [67]. Following normalization, low expressing probes (log_2_(intensity) ≤ 5) were excluded if they were expressed in less than 50%, minus one sample. BioMart was then used to annotate the probes to their corresponding target genes and only probes that successfully annotated to coding genes were retained for downstream analysis. The filtered expression matrix was subsequently loaded into the LIMMA v3.46.0 (Linear Models for Microarray data) package and differential analysis between ND and T2DM islets was performed for each dataset using the empirical Bayes procedure [68]. To combine the differential expression analysis from each of the 5 studies, we inputted the filtered analysis from LIMMA into MetaVolcanoR v1.4.0 and subsequently performed random effect modeling to assess and summarize the variance of expression for each gene between ND and T2DM islets. Subsequently, we annotated the REV-ERBα footprints unique in T2DM islets to the nearest gene loci using GREAT v4.0.4 [69] and performed a one-sample Wilcoxon test to compare the expression of these genes in ND versus T2DM islets across the 5 studies based upon the random effects model calculated above.

### Statistical analysis

Results are expressed as the means ± SEM. for *n* independent experiments, as indicated in the figure legends. Statistical analyses were carried out using Student’s *t* test or one-way analysis of variance followed by Sidak’s post hoc test for multiple comparisons using GraphPad Prism 7. A *P* value of <0.05 was taken as evidence of statistical significance (*P < 0.05, **P < 0.01, ***P < 0.001).

## Supplementary information

Supplemental Figures and Table

Supplemental file_Uncropped WB

Checklist

## Data Availability

The experimental data sets generated and/or analyzed during the current study are available from the corresponding author upon reasonable request. No applicable resources were generated during the current study.
