# Supplementary material for: The nuclear receptor REV-ERBα is implicated in the alteration of β-cell autophagy and survival under diabetogenic conditions
Source: Cell Death Dis. 2022 Apr 15;13(4):353. doi: 10.1038/s41419-022-04767-z (PMC9012816; doi:10.1038/s41419-022-04767-z)
Supplement: Supplementary file 1 — Supplemental Figures and Table [file 41419_2022_4767_MOESM1_ESM.pdf]

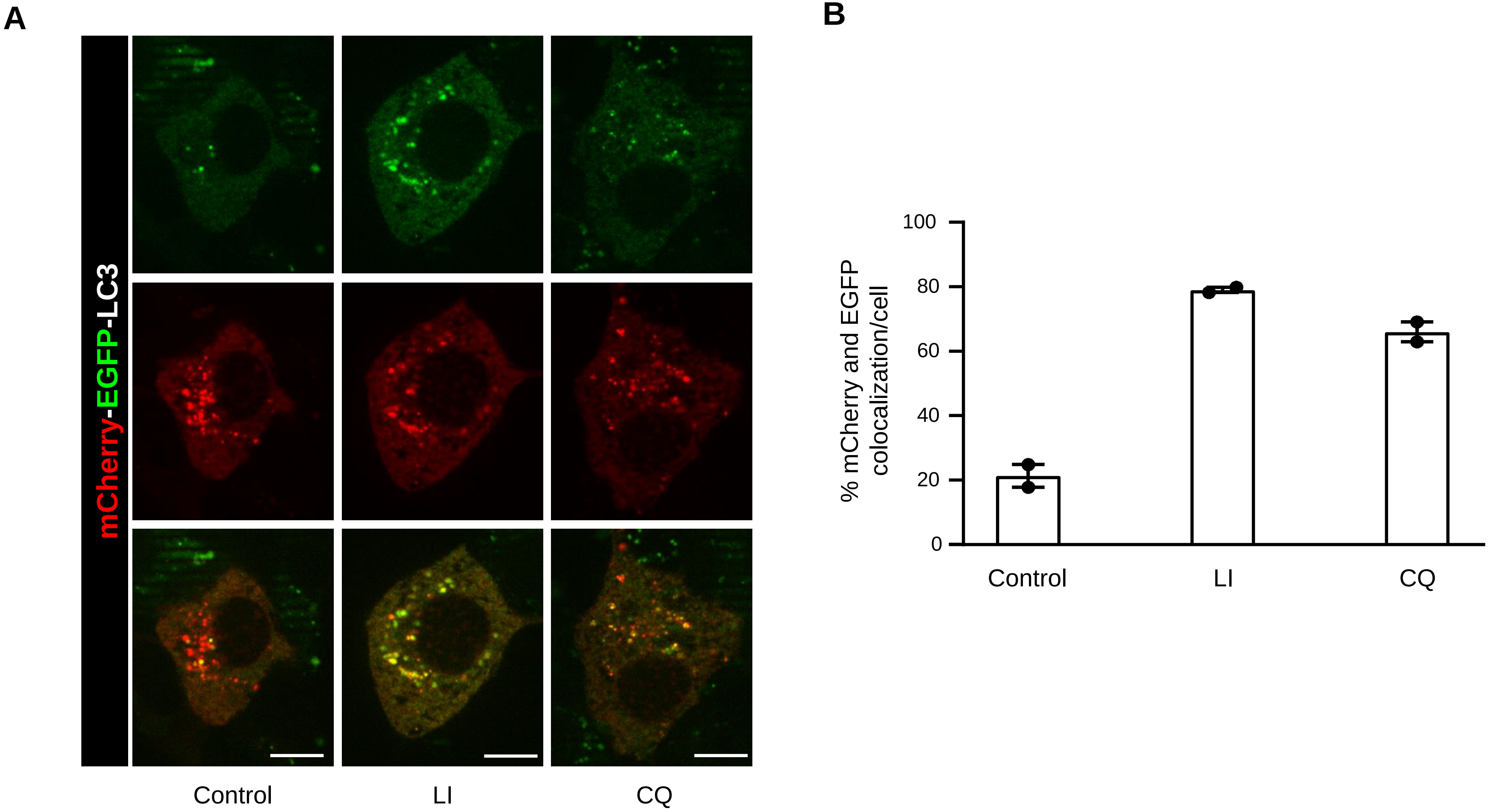


**<u>Supplemental Figure 1.</u> Validation of mCherry-EGFP-LC3 reporter system in β-cells.**
**A:** INS-1E cells expressing mCherry-EGFP-LC3B plasmid were either cultured in the absence or presence of lysosomal inhibitors (LI: E-64-d, 10 μg/ml and pepstatin A, 10 μg/ml, 24 h), or chloroquine (CQ: 100 μM, 2 h). **B:** Autophagic flux was assessed by quantification of mCherry and EGFP puncta colocalization using Image J software. Results are expressed as percentage of mCherry and EGFP colocalization per cell. Data are expressed as mean ± SEM of two independent experiments. Scale bar: 5 μm

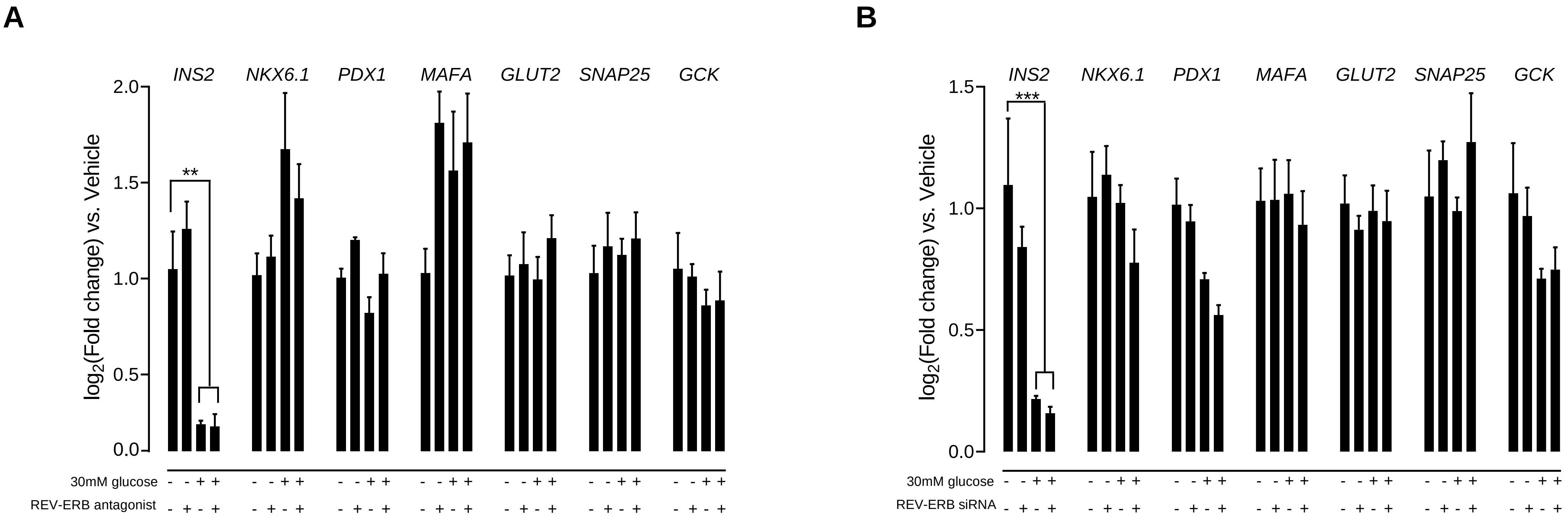


**<u>Supplemental Figure 2:</u> Role of REV-ERBα silencing or inhibition on β-cell function and identity gene expression under glucotoxic conditions.**
**A:** INS-1E cells were exposed to glucotoxicity [30 mM glucose (G30) vs. control 11 mM glucose (G11)] for 48 h in the presence or absence of REV-ERB antagonist (SR8279, 10 μM). **B:** REV-ERBα was silenced in INS-1E cells by siRNA. Scramble RNA (-) was used as control. Cells were then exposed to glucotoxicity for 48 h (G30 vs. G11). For **A** and **B**, mRNA expression of key β-cell identity and functional genes is plotted and expressed as fold change relative to control expression (n=4 independent experiments per condition). Data are expressed as mean ± SEM; **P<0.01, ***P<0.001

| Gene Target (Rat) | Sequence (F=forward; R=reverse) |
|---|---|
| *Ins2* | F-ATCCTCTGGGAGCCCCGC |
| | R-AGAGAGCTTCCACCAAG |
| *Nkx6.1* | F-CAGGTCAAGGTCTGGTTCCA |
| | TCAGTCTCCGAGTCCTGCT |
| *Pdx1* | F-GAACCGGAGGAGAATAAGAGG |
| | R-AGTCAAGTTGAGCATCACTGC |
| *Mafa* | F-AGGAGGTCATCCGACTGAAACA |
| | R-GCGTAGCCGCGGTTCTT |
| *Glut2 (Slc2a2)* | F-CGGAACCTTGGCTTTCACTGTCTT |
| | R-GGTGCATTGATCACACCGATGTCA |
| *Snap25* | F-GAGTCCCTGGAAAGCACC |
| | R-GGCATCGTTTGTTACCCT |
| Gck | F-AAGCCGCAGTGAGGACGTGATG |
| | R-AGGTGATTTCGCAGTTGGGTGTCA |
| *Actin* | F-CTCCTAGCACCATGAAGATC |
| | R-TCATCGTACTCCTGCTTGC |

**Supplemental Table 1: Gene Specific Primers**
