# Supplementary material for: The nuclear receptor REV-ERBα is implicated in the alteration of β-cell autophagy and survival under diabetogenic conditions
Source: Cell Death Dis. 2022 Apr 15;13(4):353. doi: 10.1038/s41419-022-04767-z (PMC9012816; doi:10.1038/s41419-022-04767-z)

Full and uncropped western blot for Figure 1A
Lanes 1, 2 are on the figure

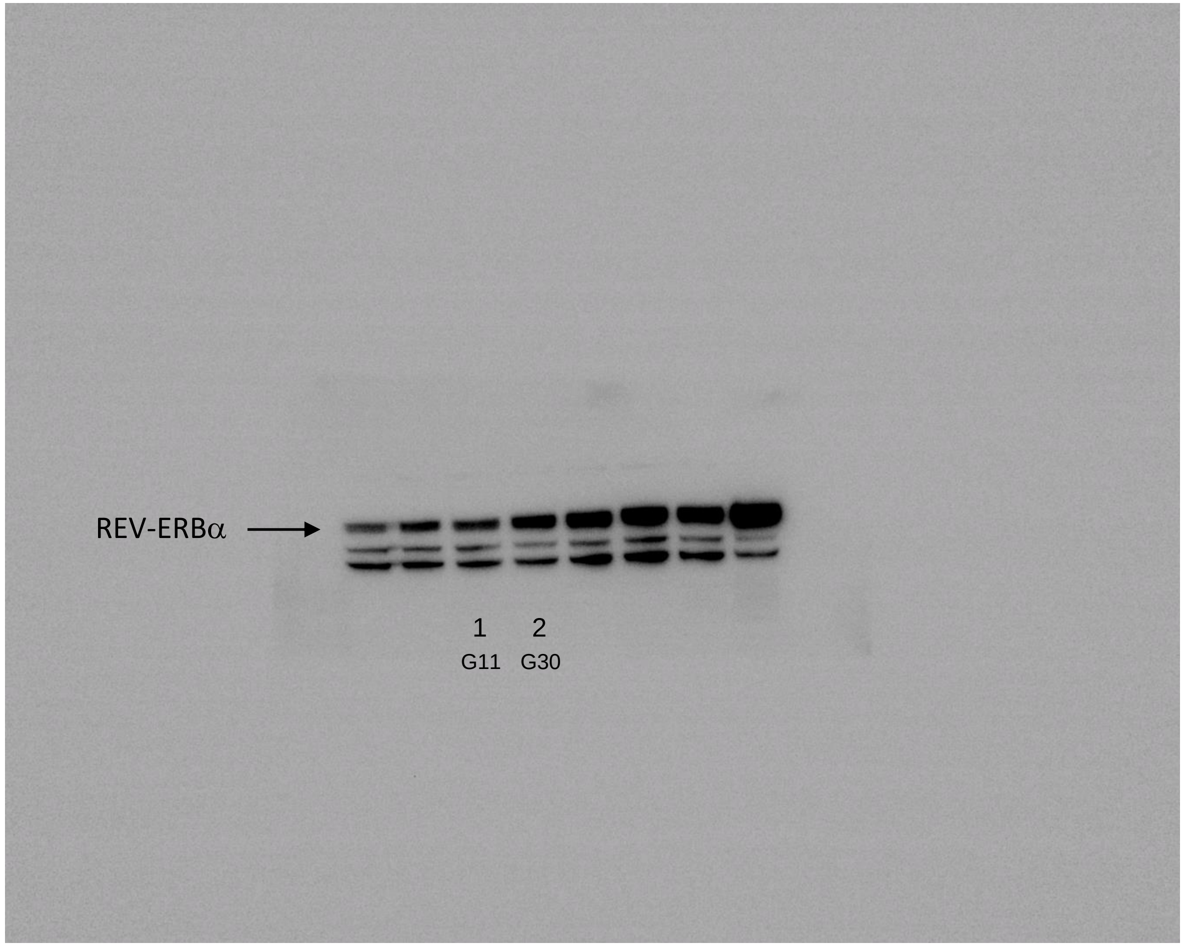

Full and uncropped western blot for Figure 1A
Lanes 1, 2 are on the figure

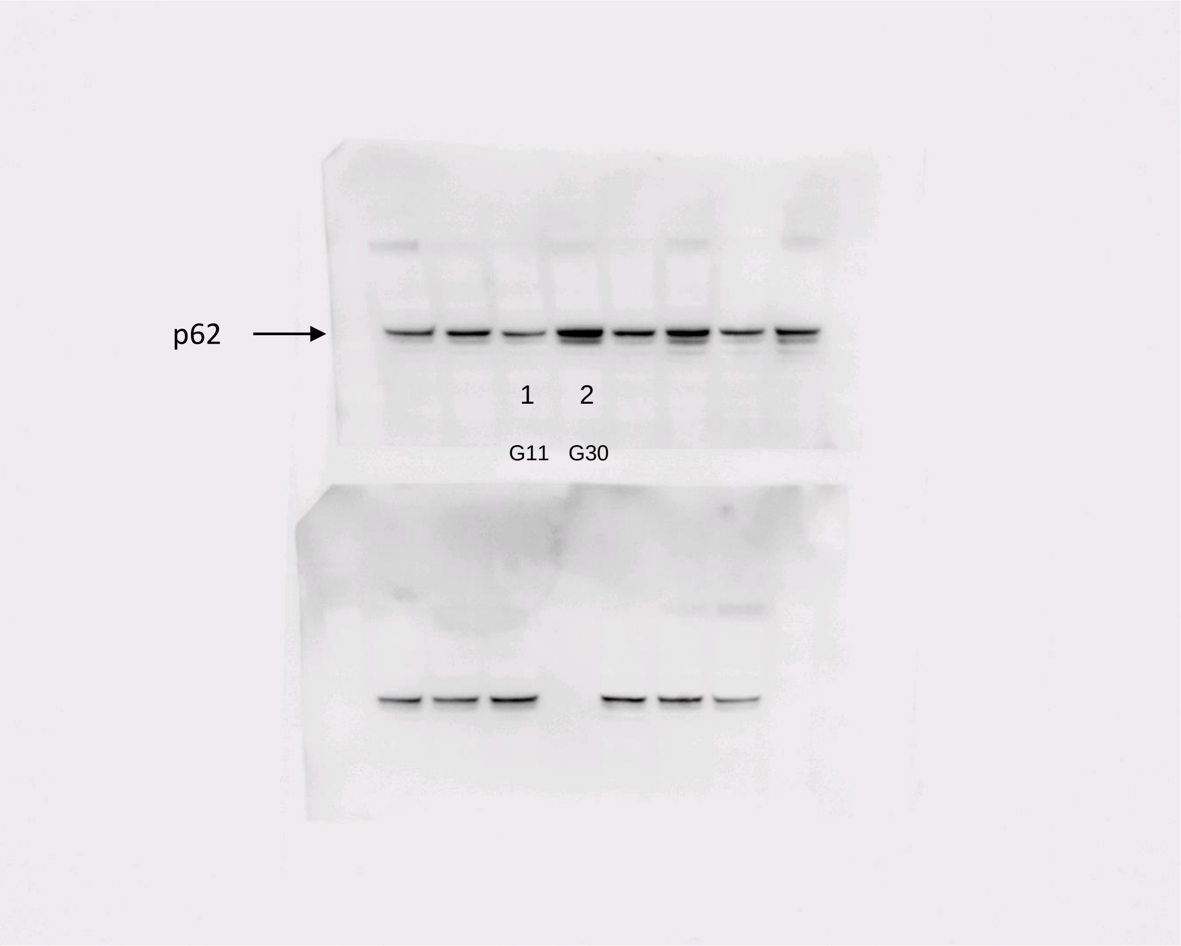

Full and uncropped western blot for Figure 1A

Lanes 1, 2 are on the figure

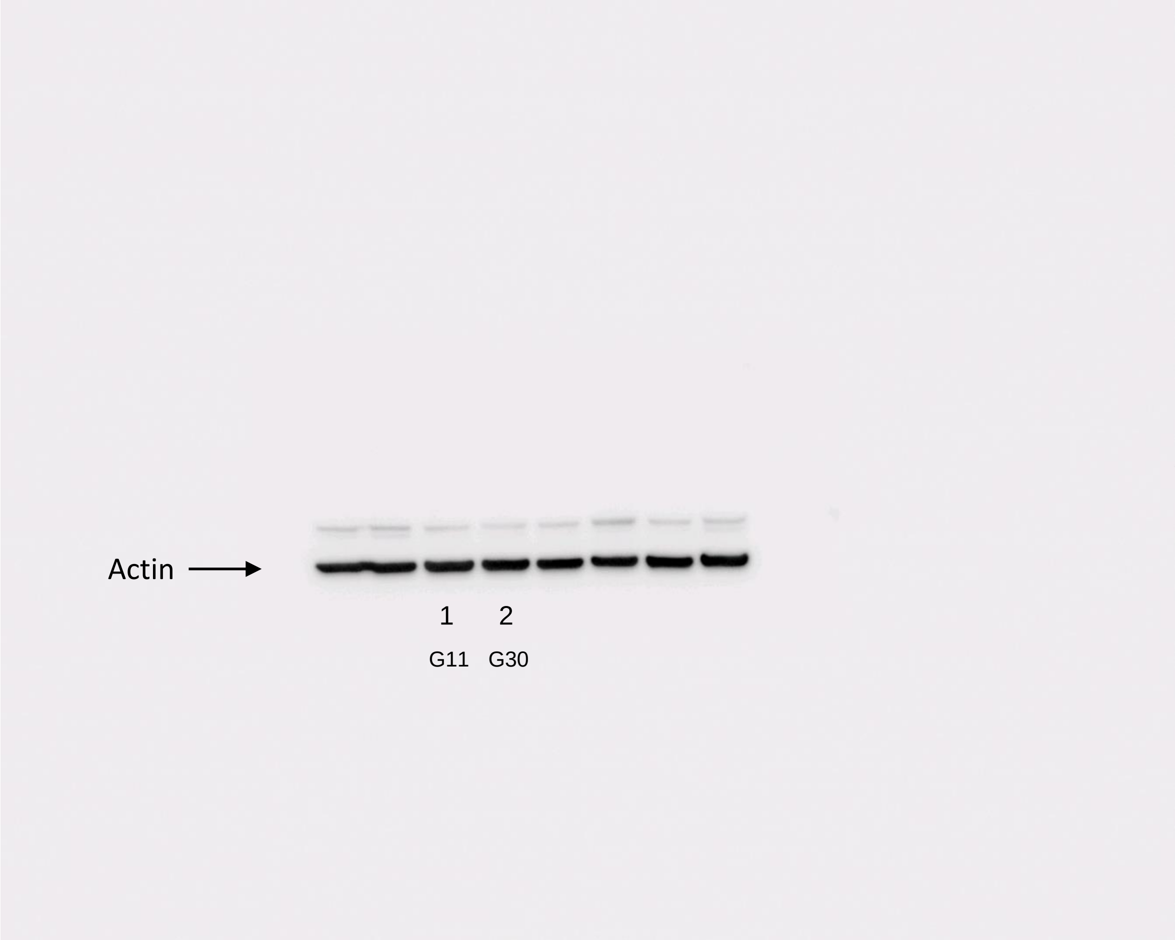

Full and uncropped western blot for Figure 1A
Lanes 1, 2 are on the figure

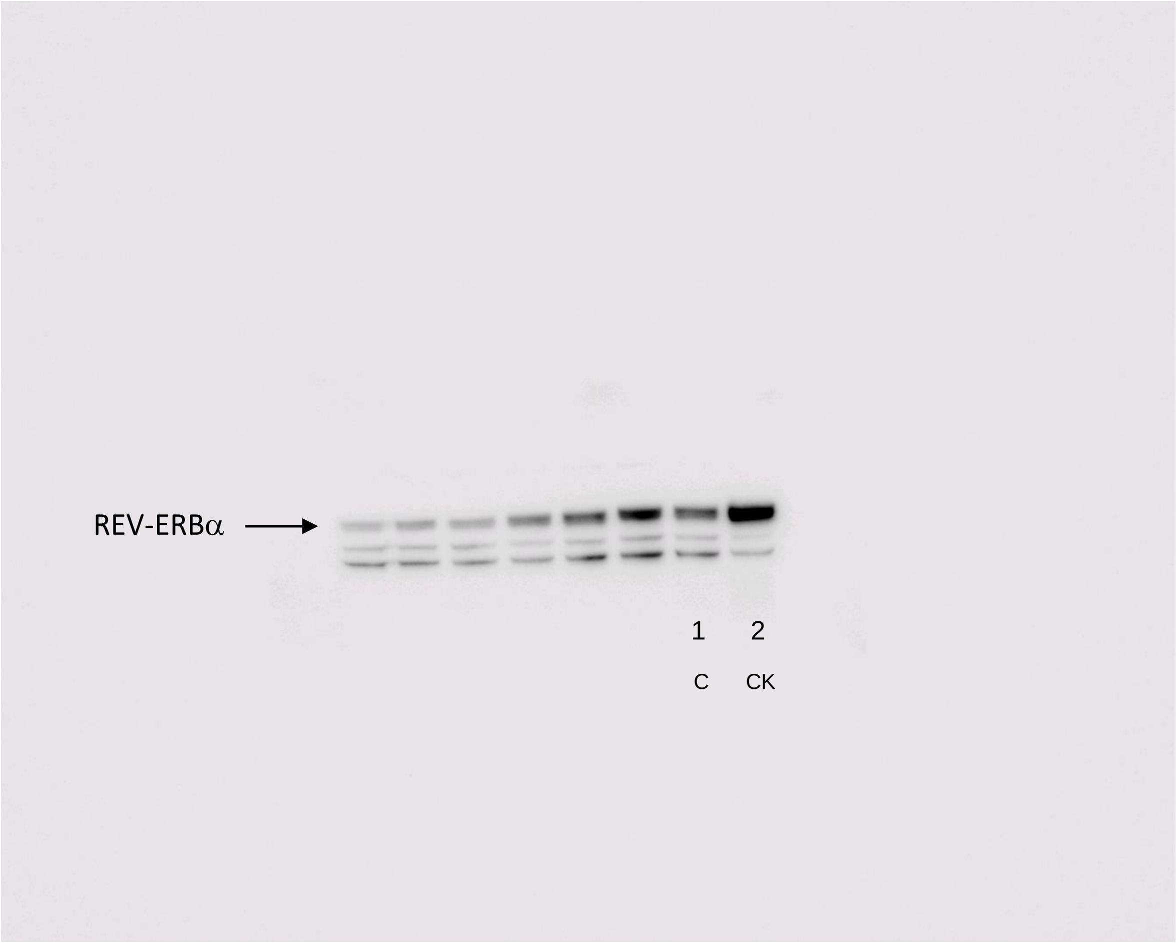

Full and uncropped western blot for Figure 1A
Lanes 1, 2 are on the figure

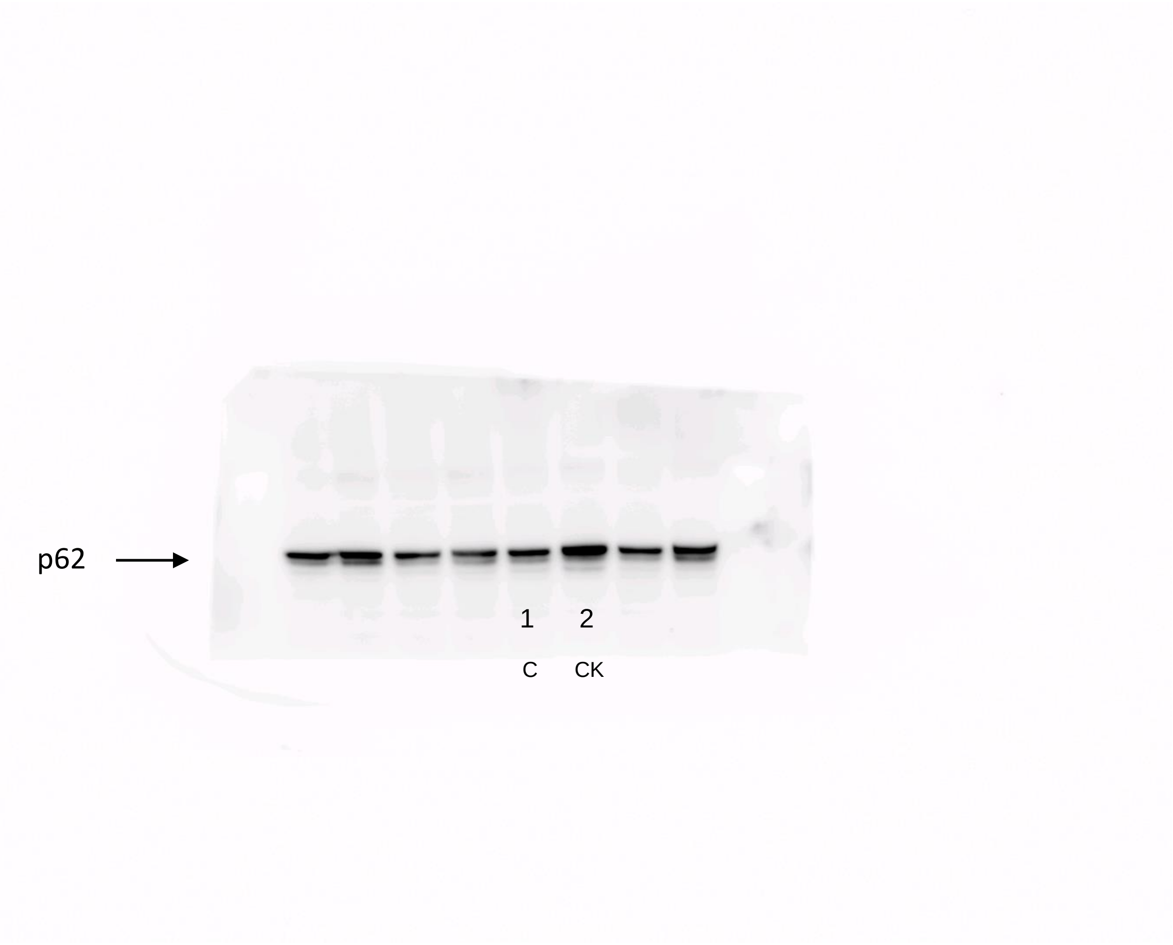

Full and uncropped western blot for Figure 1A

Lanes 1, 2 are on the figure

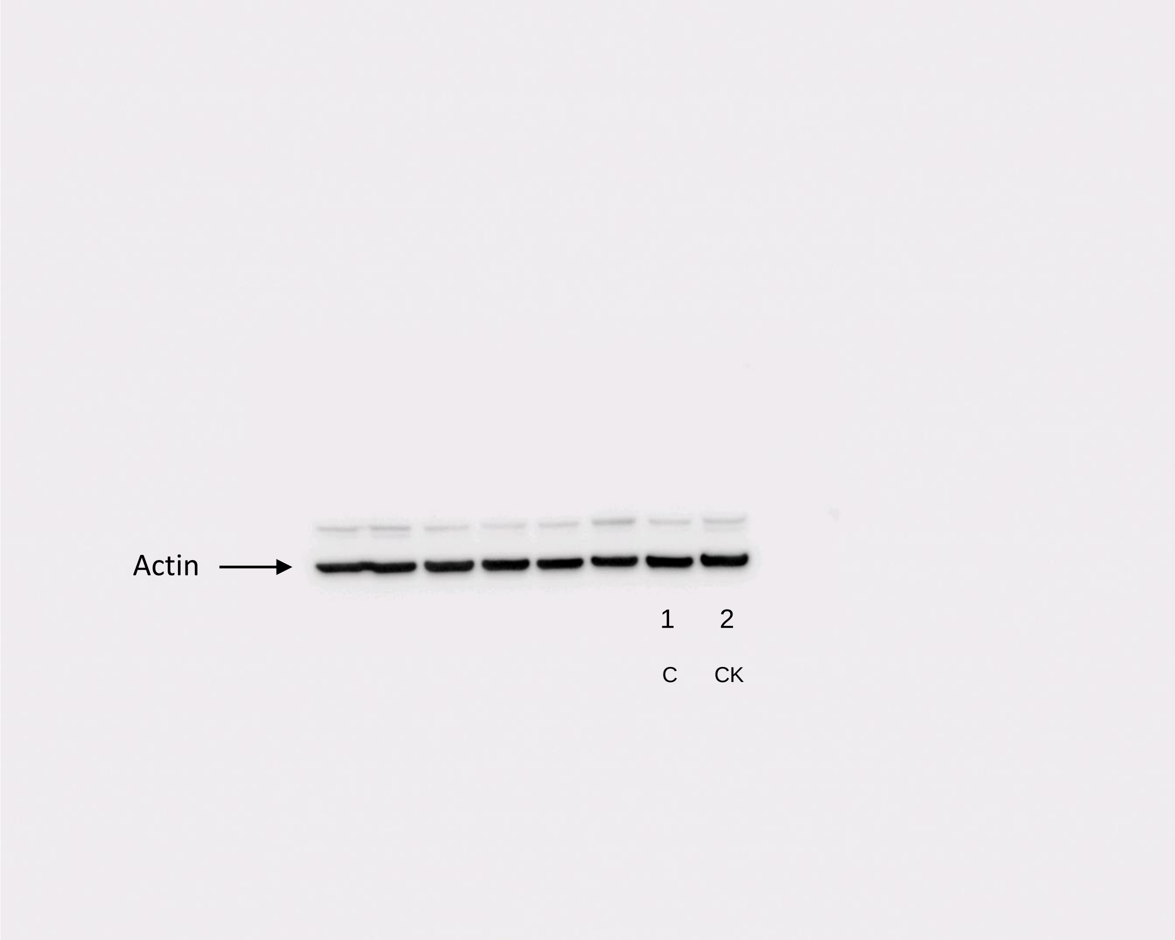

Full and uncropped western blot for Figure 1C

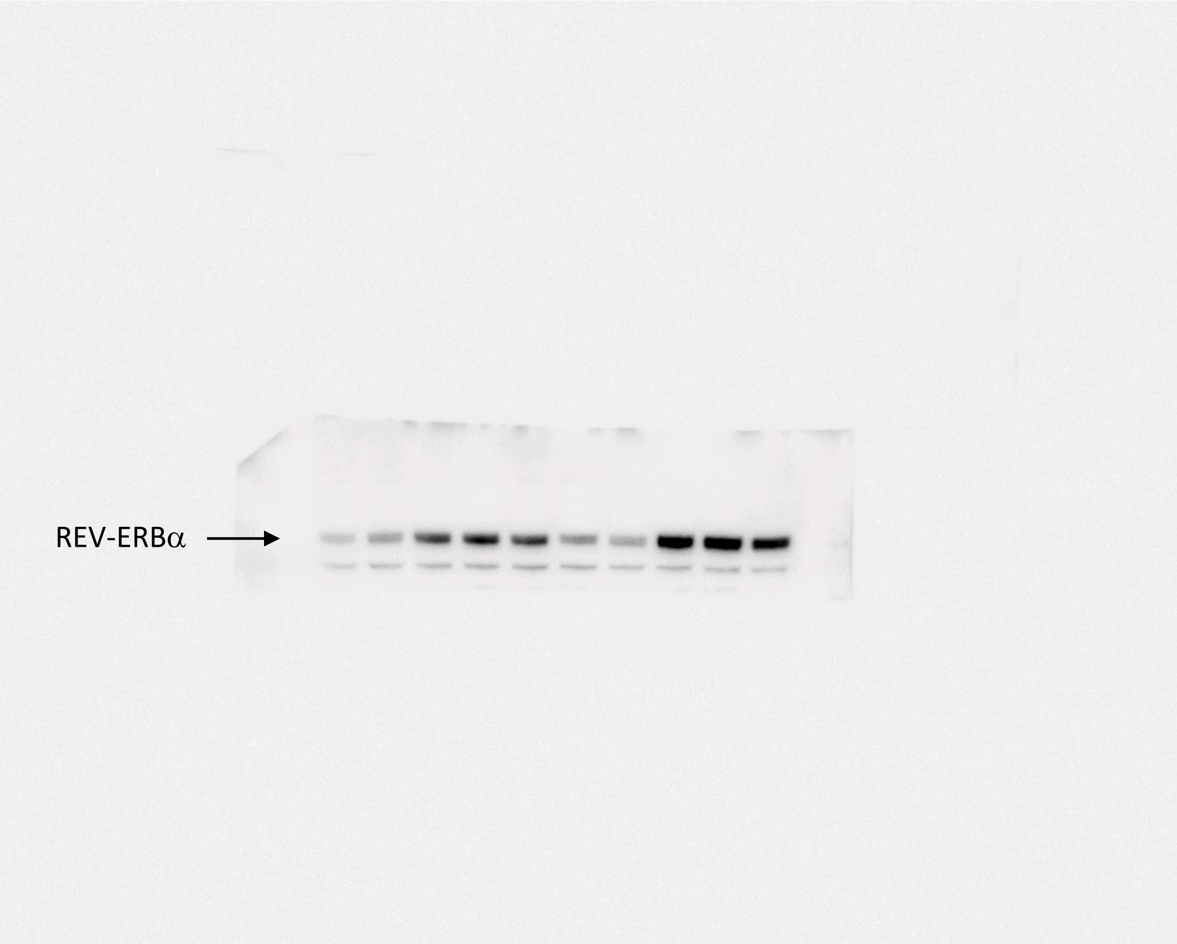

Full and uncropped western blot for Figure 1C

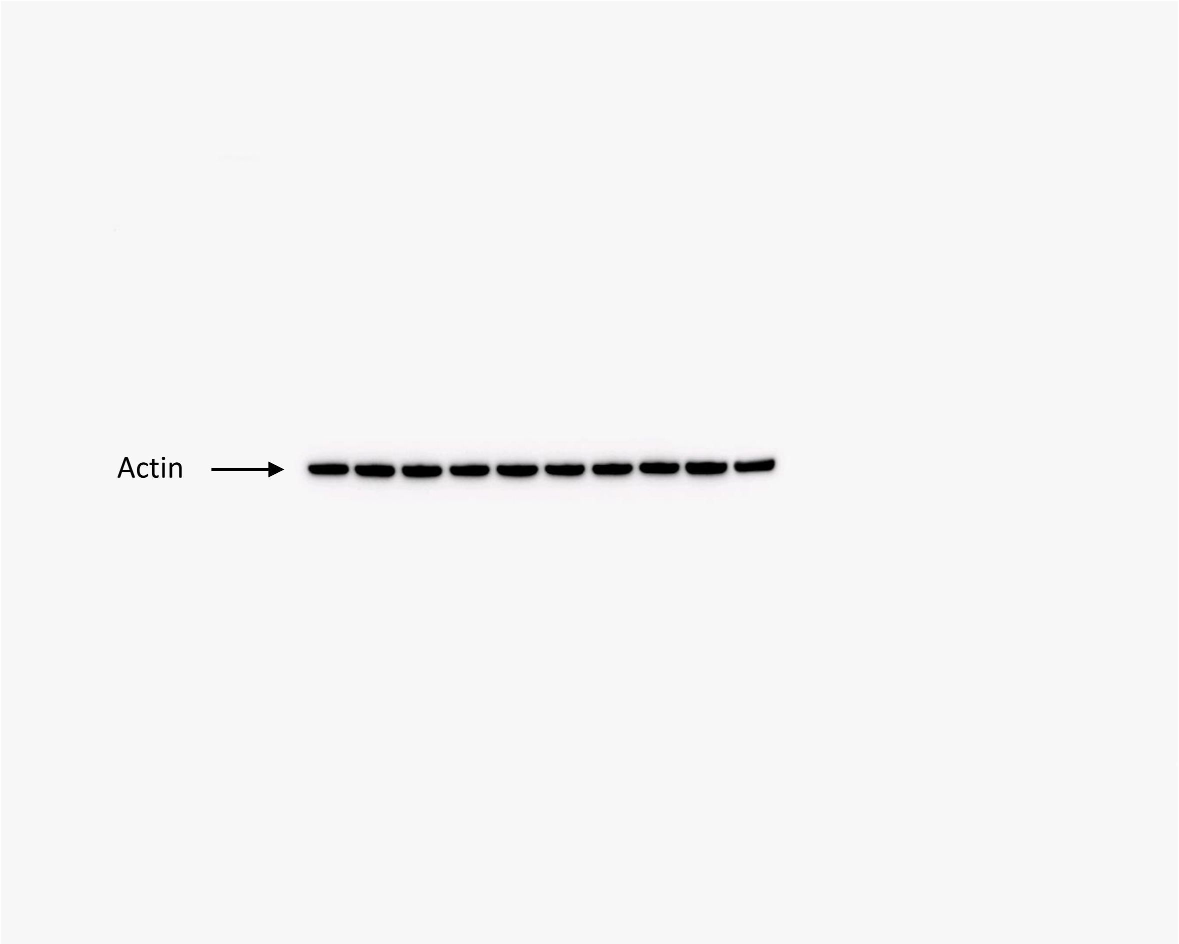

Full and uncropped western blot for Figure 2A
Lanes 1, 2, 3 are on the figure

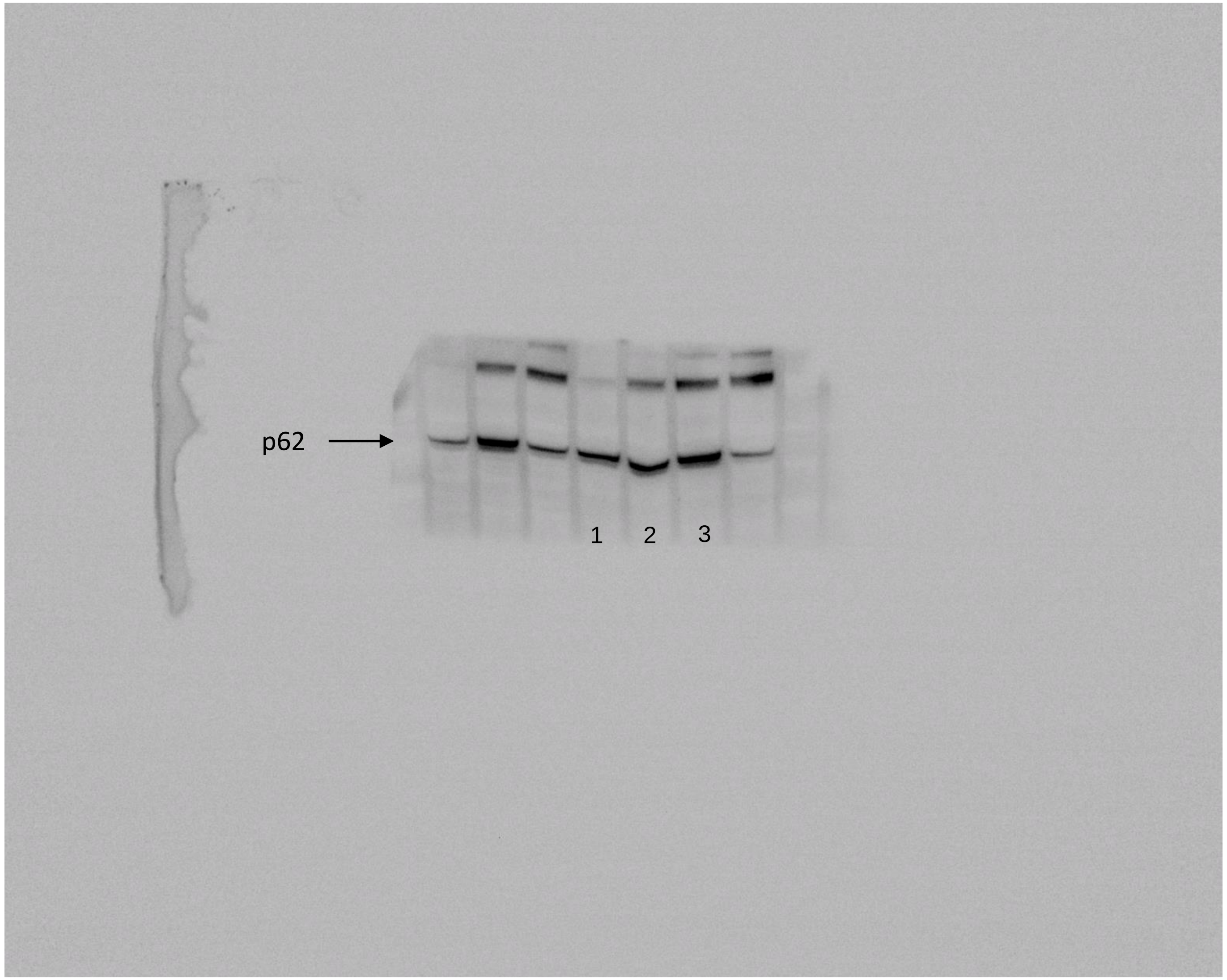

Full and uncropped western blot for Figure 2A

Lanes 1, 2, 3 are on the figure

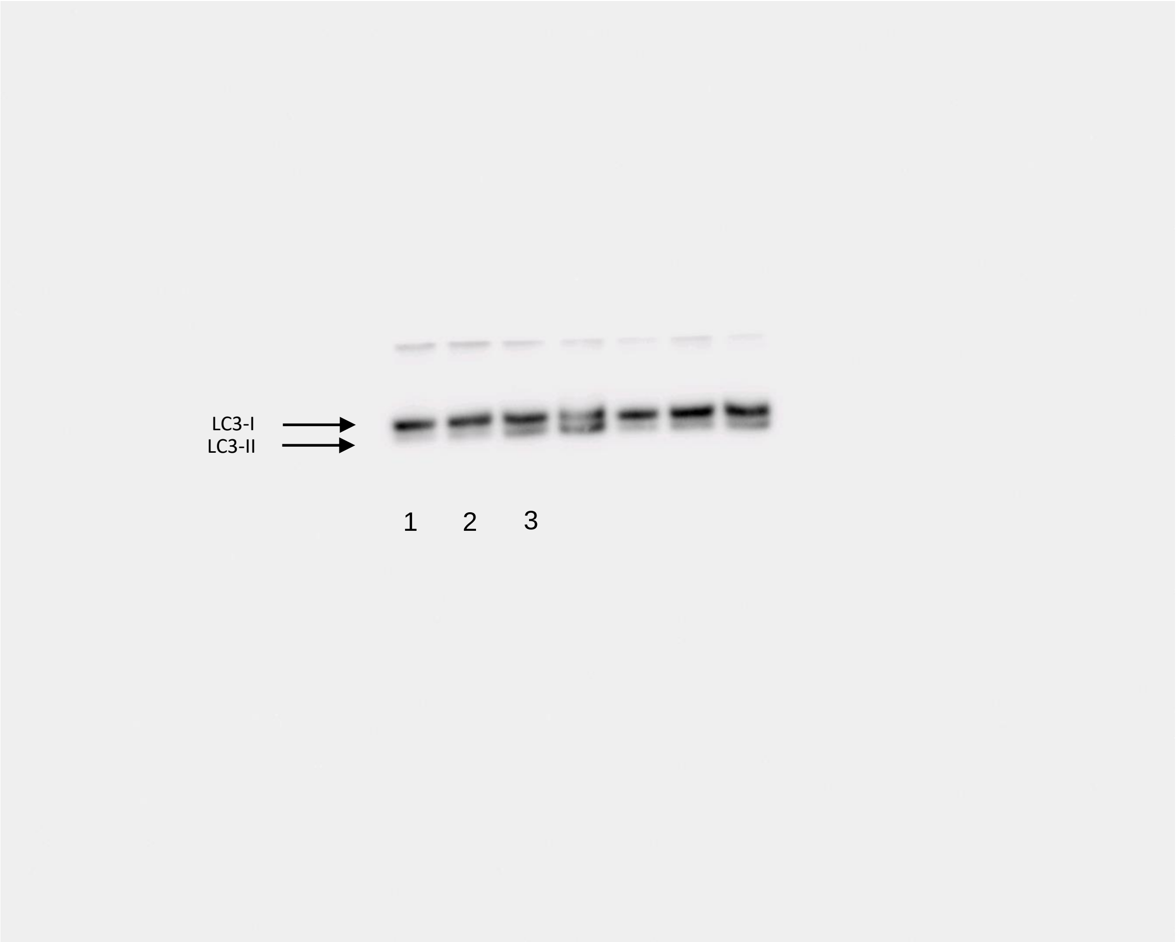

Full and uncropped western blot for Figure 2A

Lanes 1, 2, 3 are on the figure

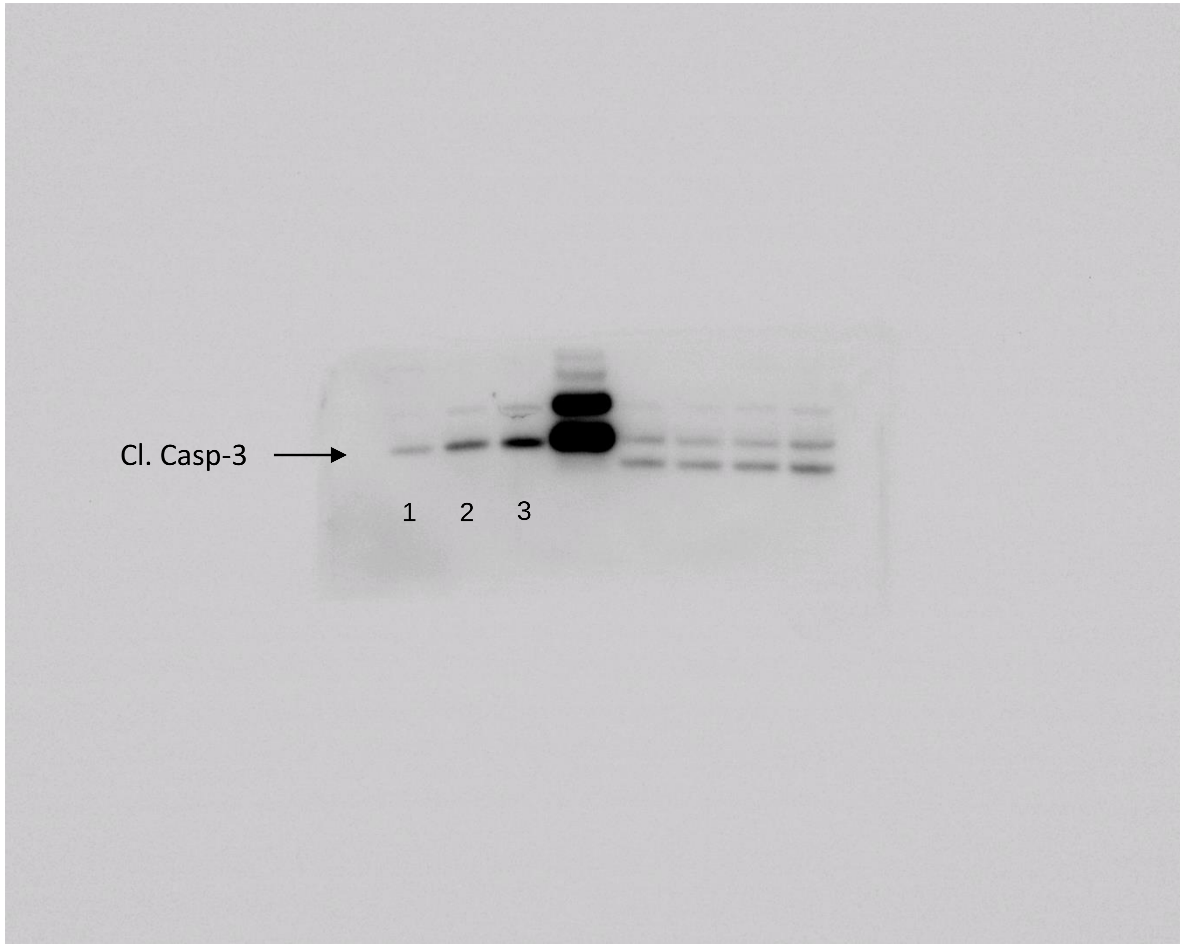

Full and uncropped western blot for Figure 2A

Lanes 1, 2, 3 are on the figure

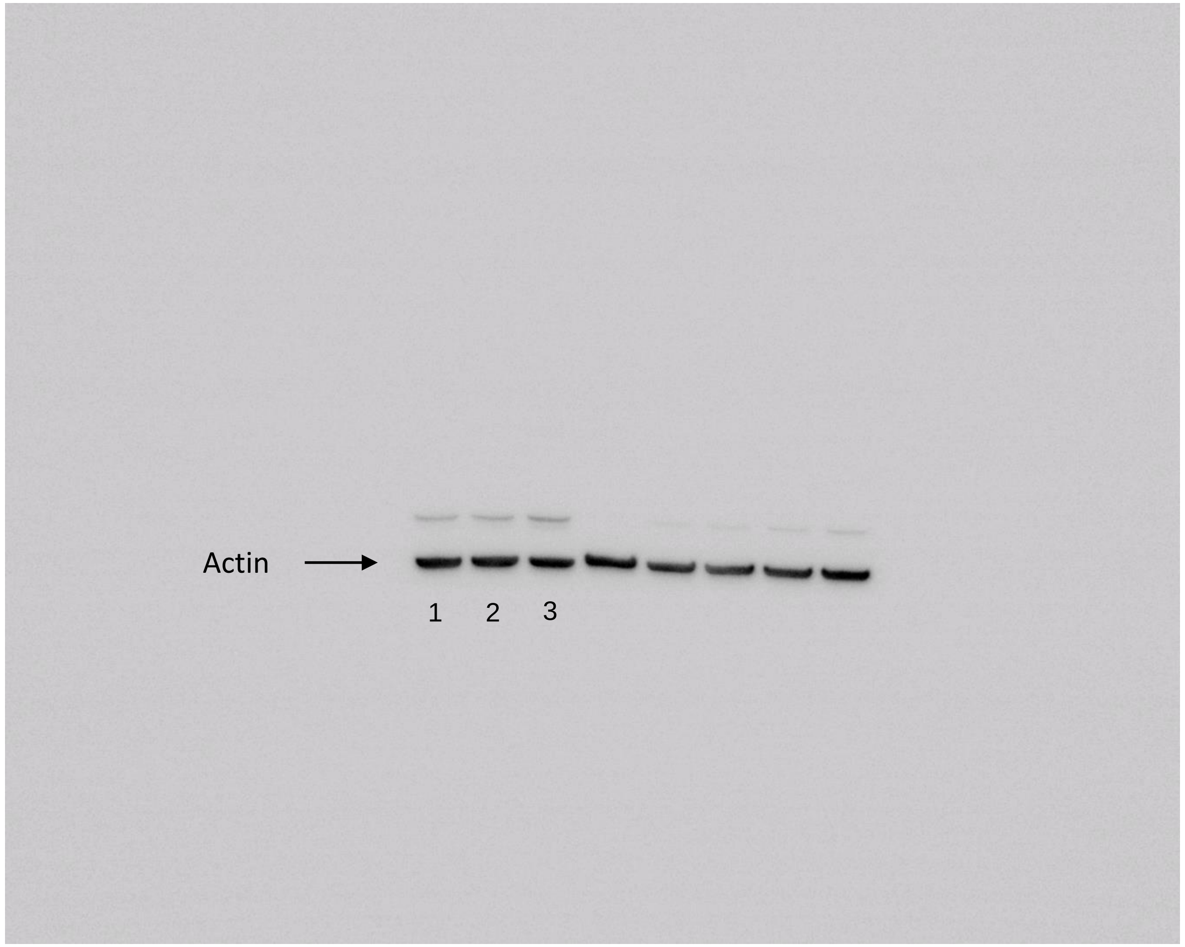

Full and uncropped western blot for Figure 2C

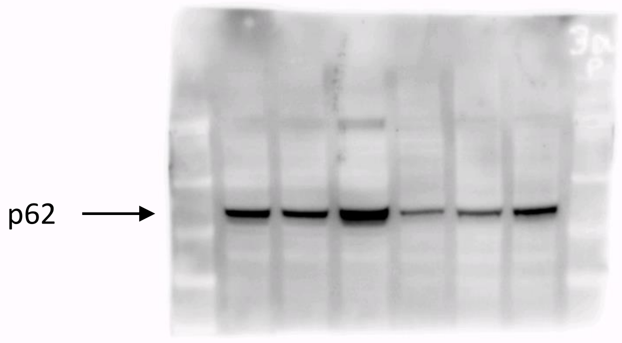

Full and uncropped western blot for Figure 4A
Lanes 1, 2 are on the figure

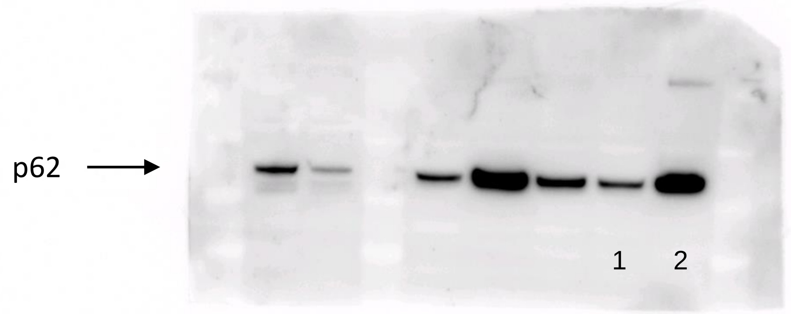

Full and uncropped western blot for Figure 4A

Lanes 1, 2 are on the figure

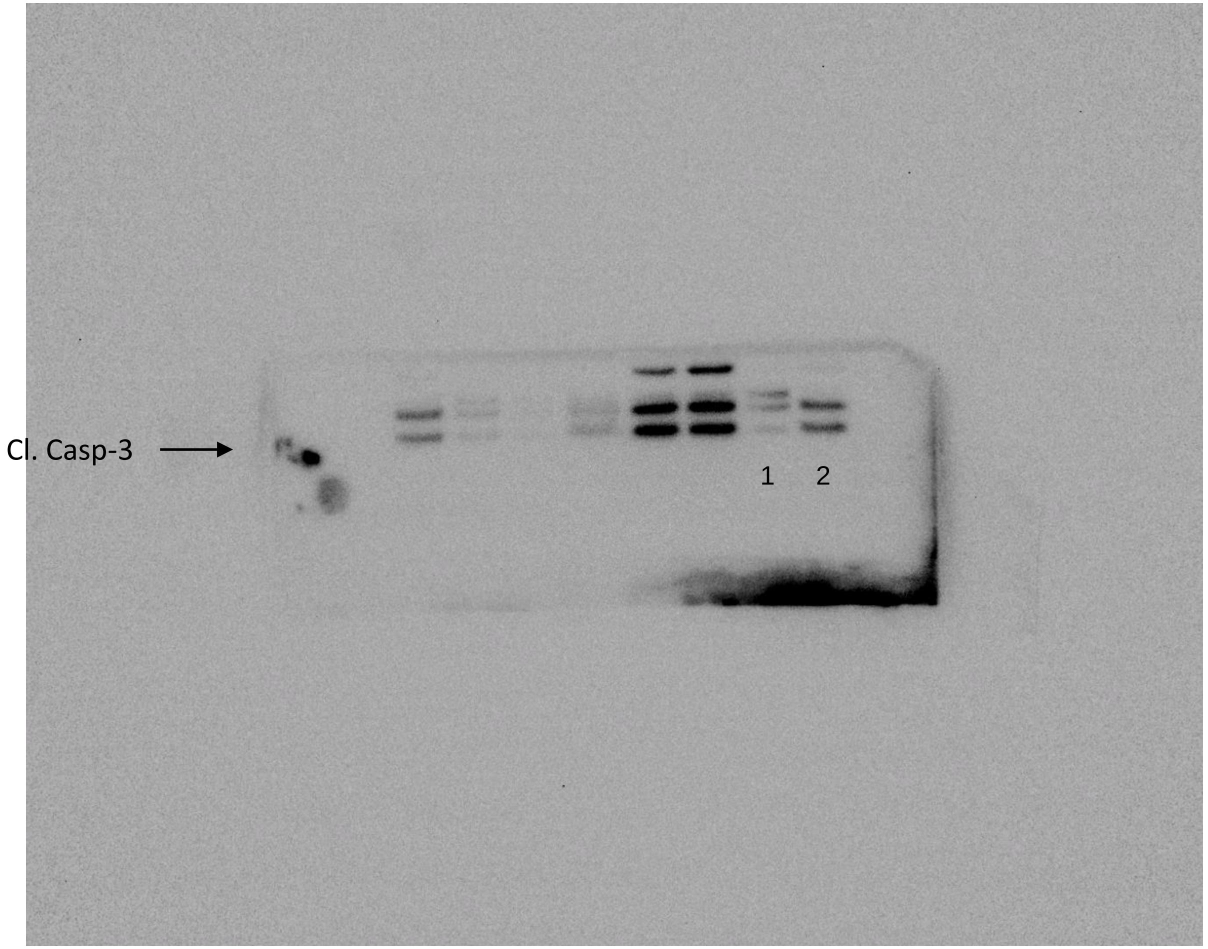

Full and uncropped western blot for Figure 4A

Lanes 1, 2 are on the figure

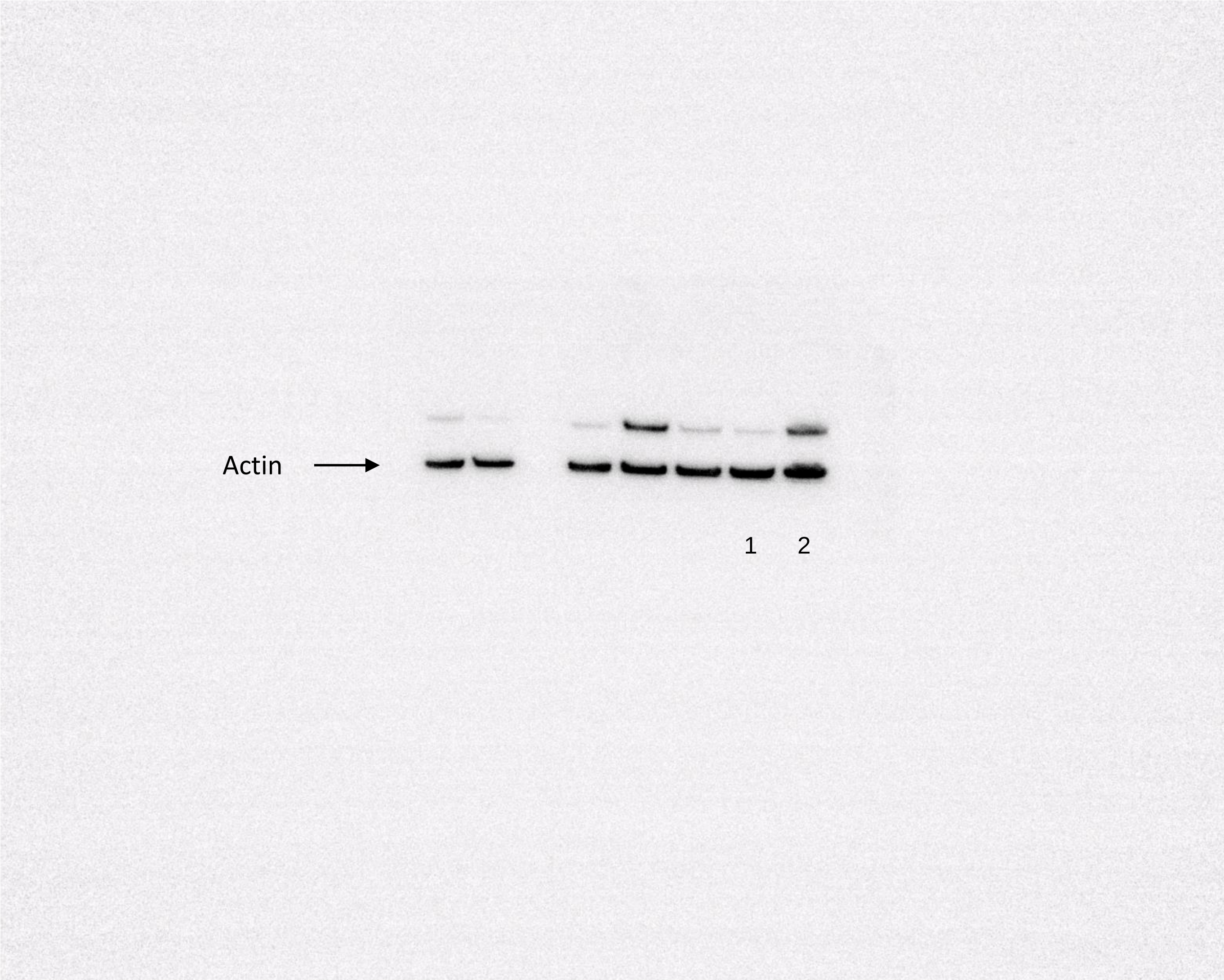

Full and uncropped western blot for Figure 6A

Lanes 1, 2, 3, 4 are on the figure

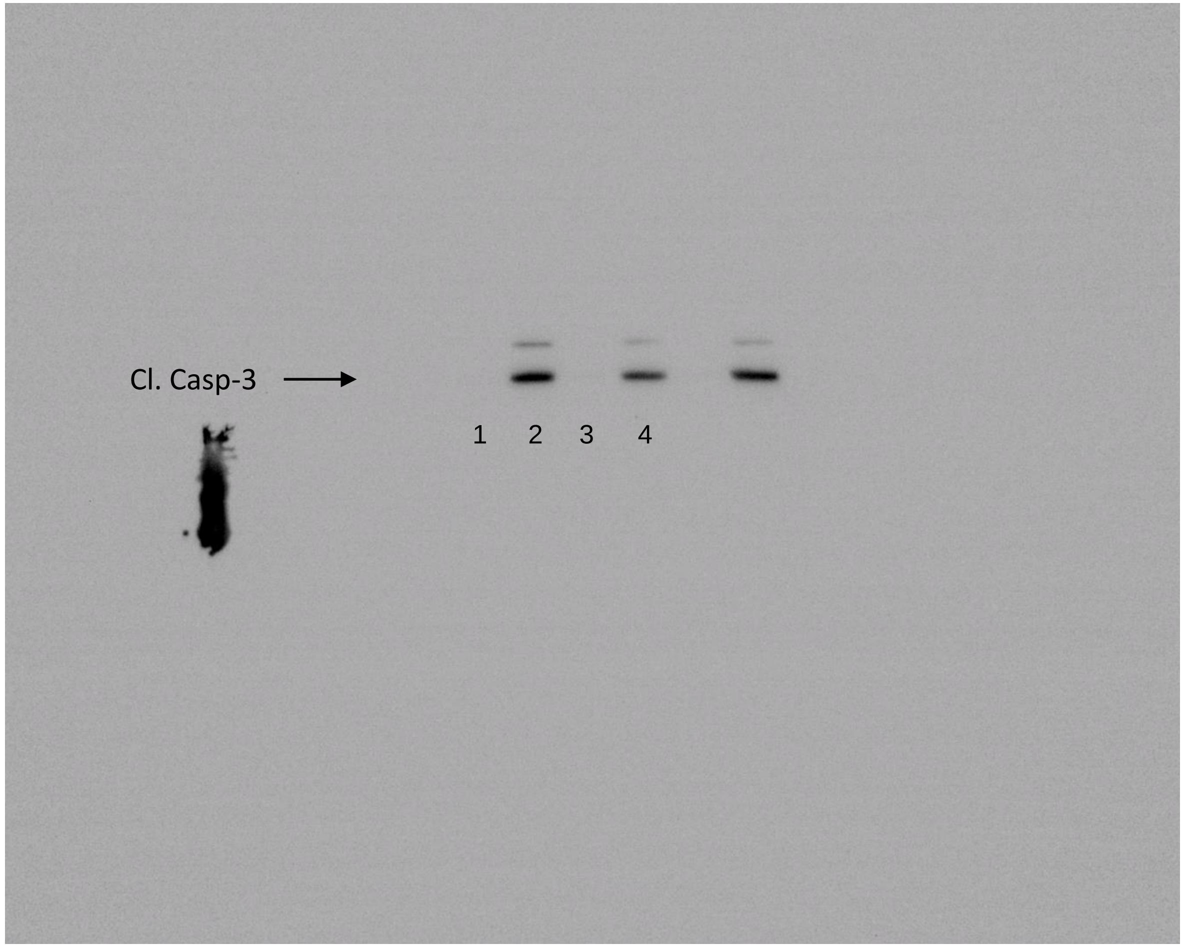

Full and uncropped western blot for Figure 6A

Lanes 1, 2, 3, 4 are on the figure

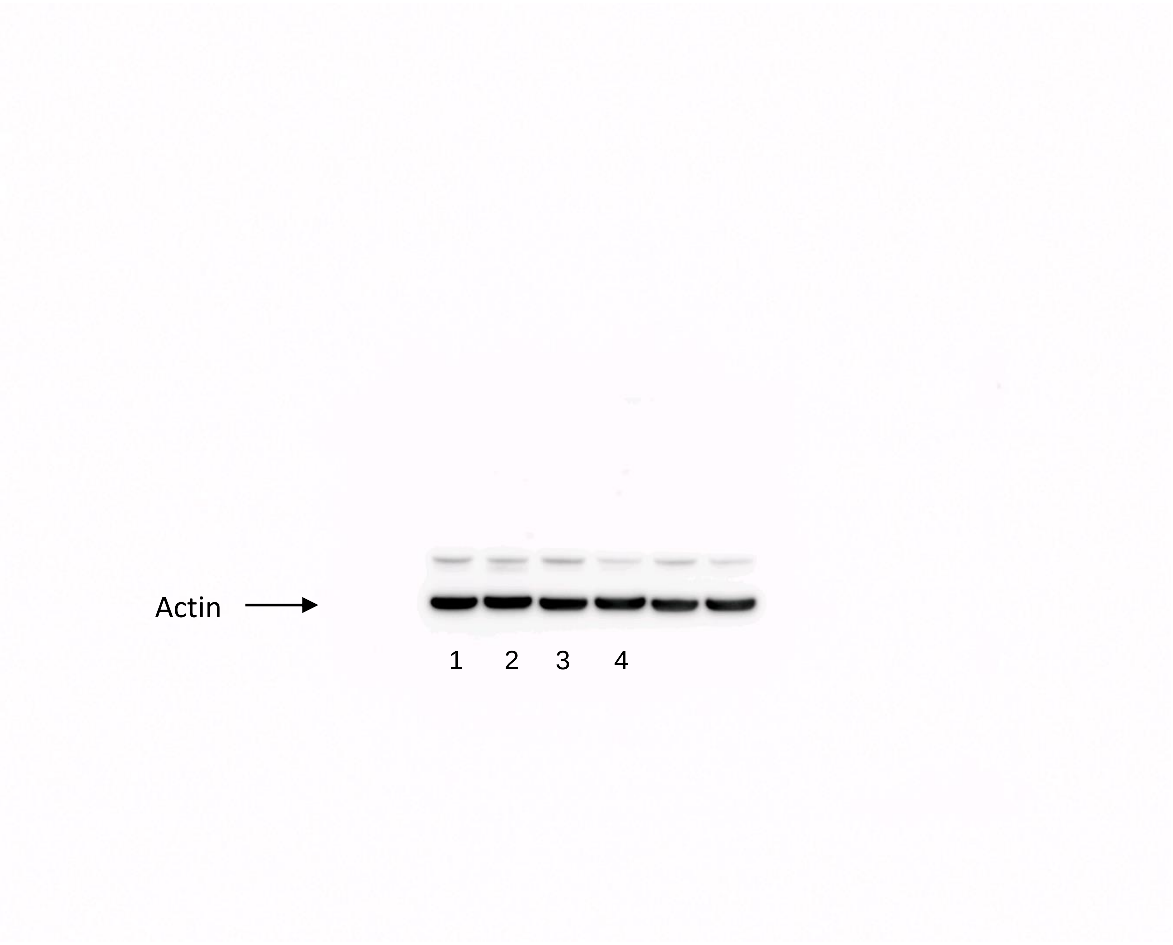

Full and uncropped western blot for Figure 6B

Lanes 1, 2, 3 are on the figure

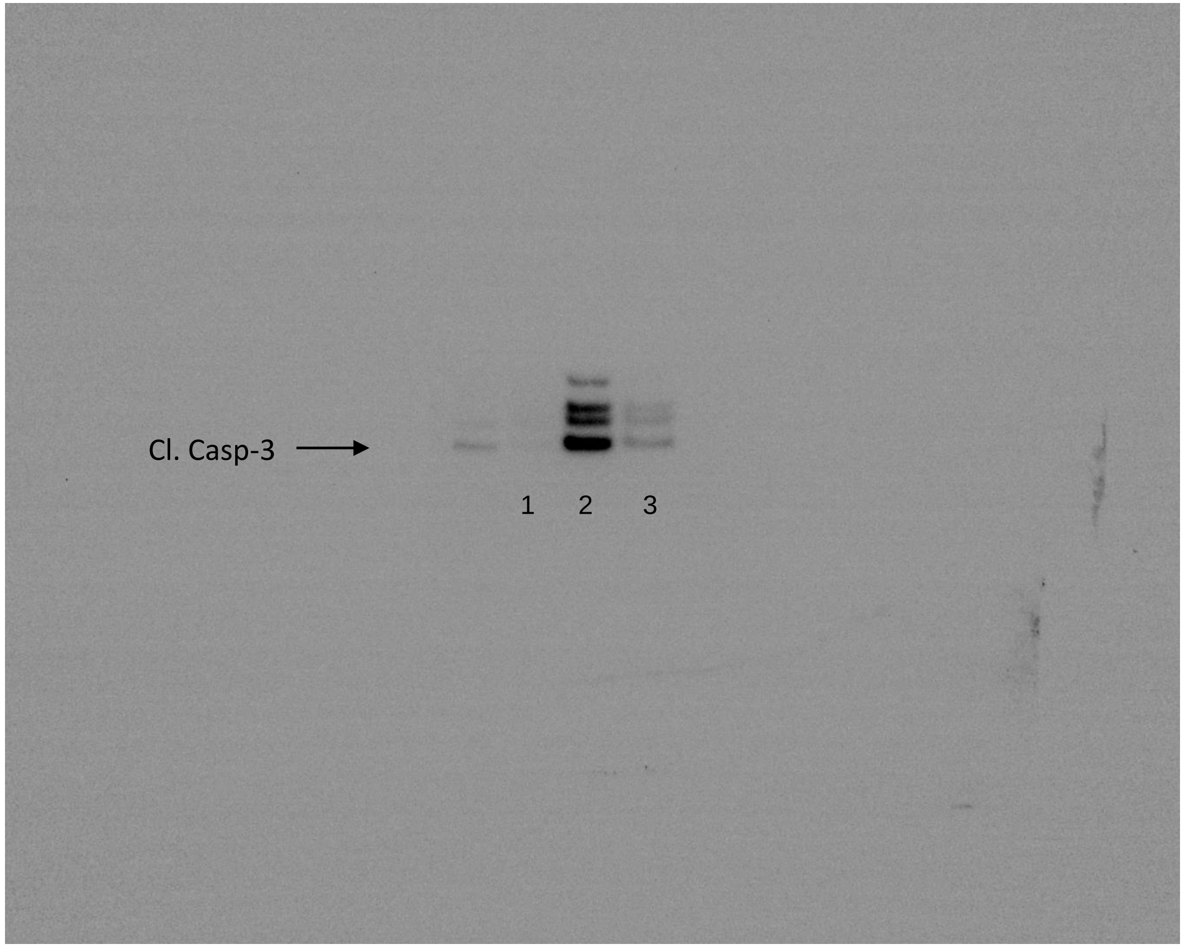

Full and uncropped western blot for Figure 6B
Lanes 1, 2, 3 are on the figure

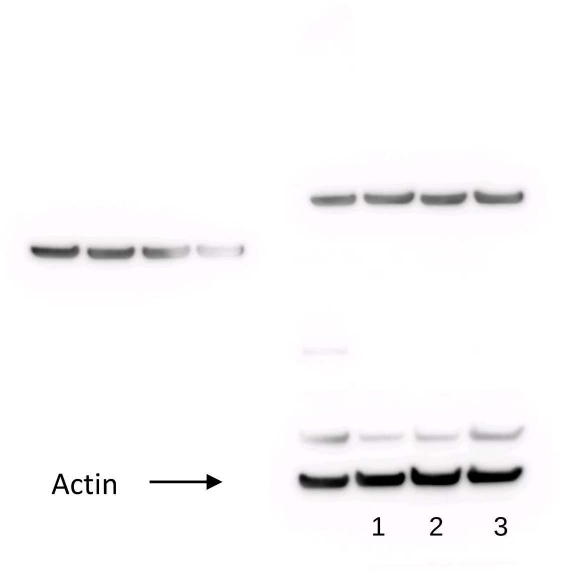

Full and uncropped western blot for Figure 6C
Lanes 1, 2, 3, 4 are on the figure

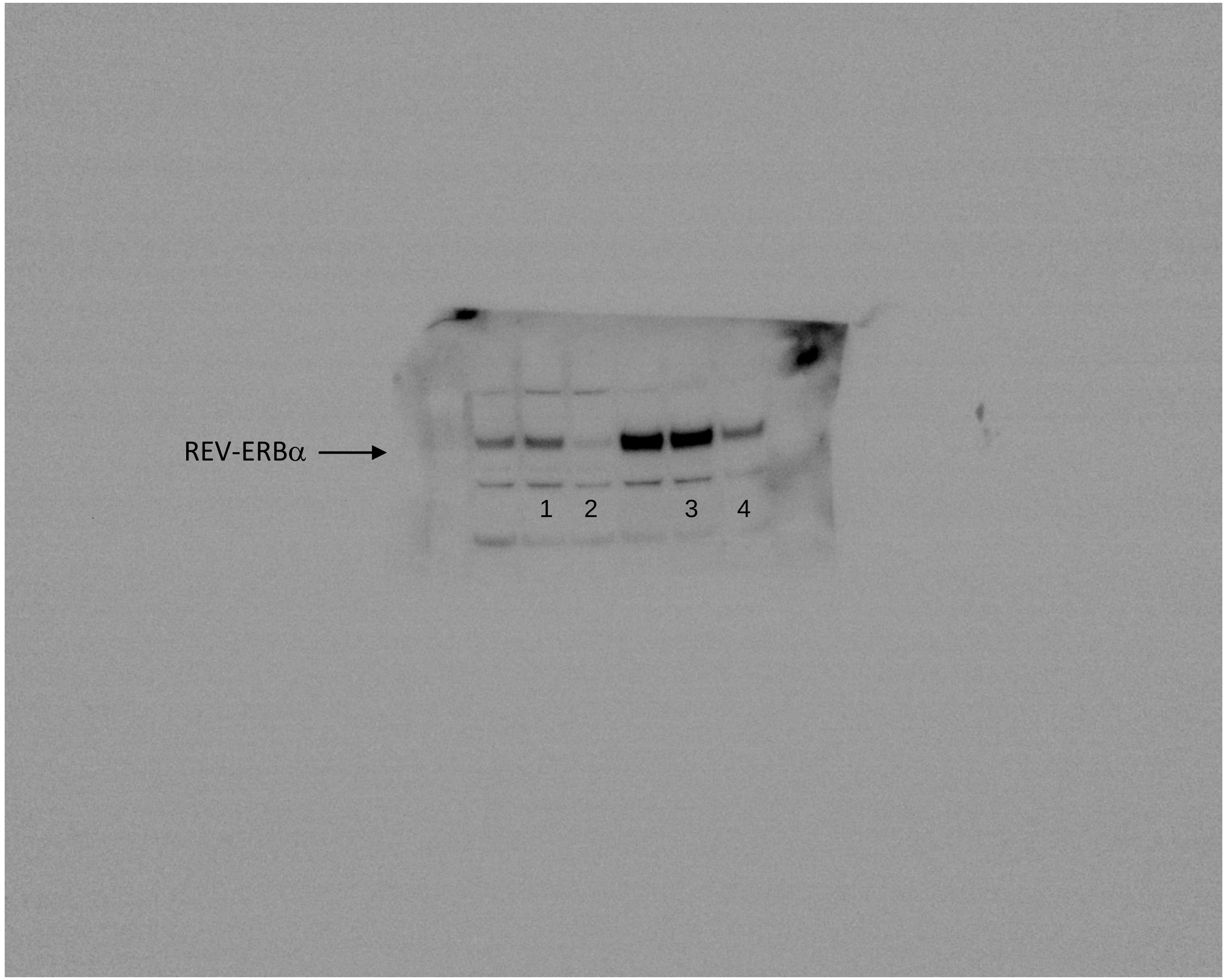

Full and uncropped western blot for Figure 6C

Lanes 1, 2, 3, 4 are on the figure

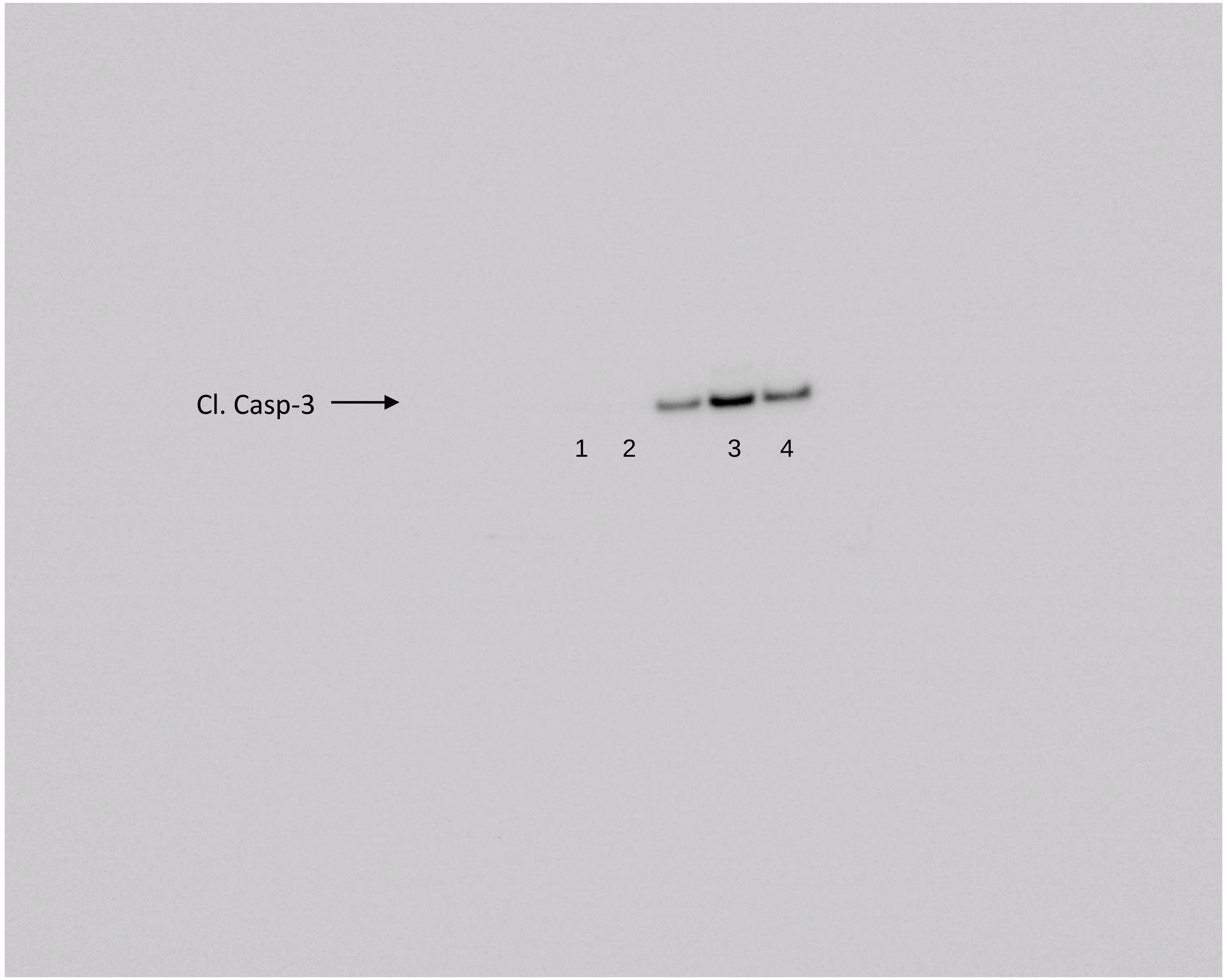

Full and uncropped western blot for Figure 6C

Lanes 1, 2, 3, 4 are on the figure

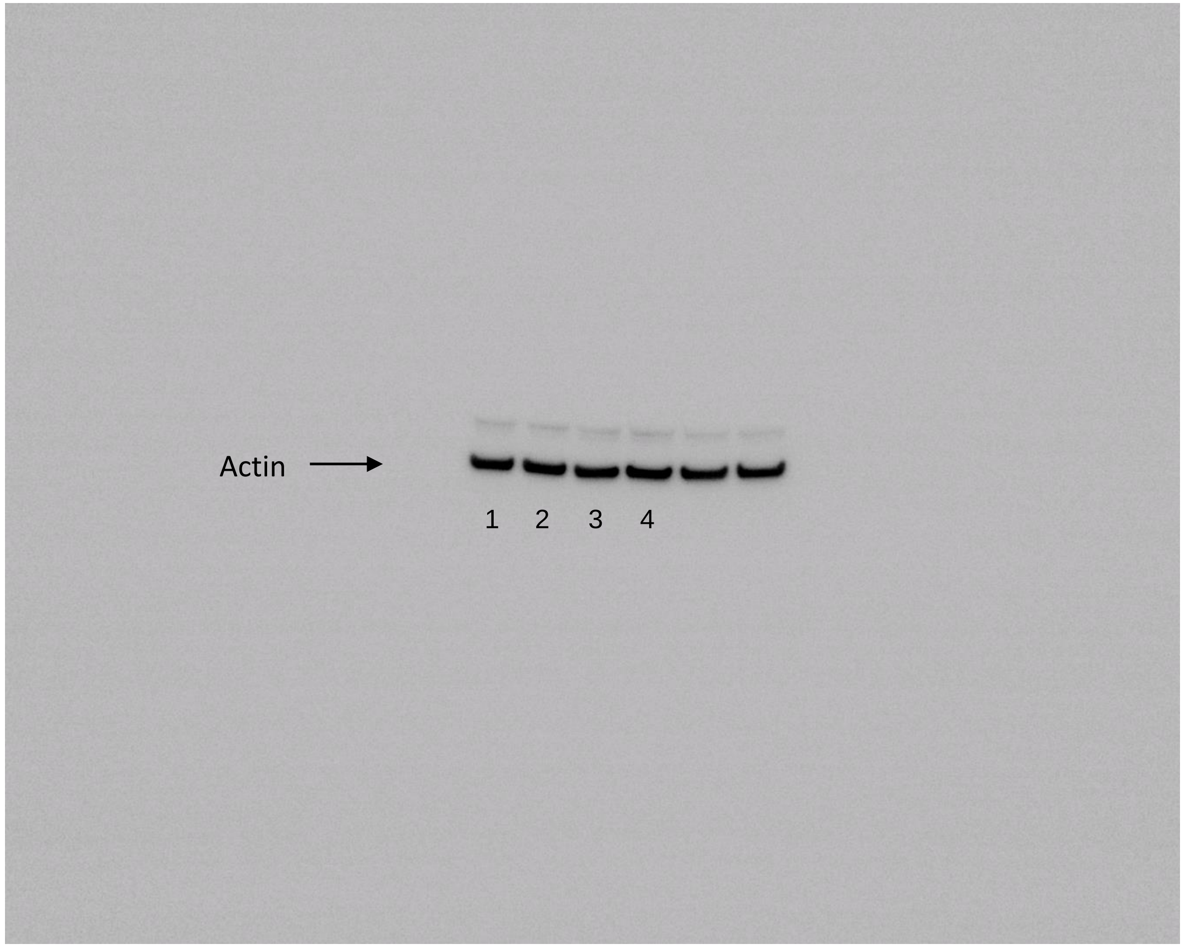

Full and uncropped western blot for Figure 7A
Lanes 1, 2, 3, 4 are on the figure

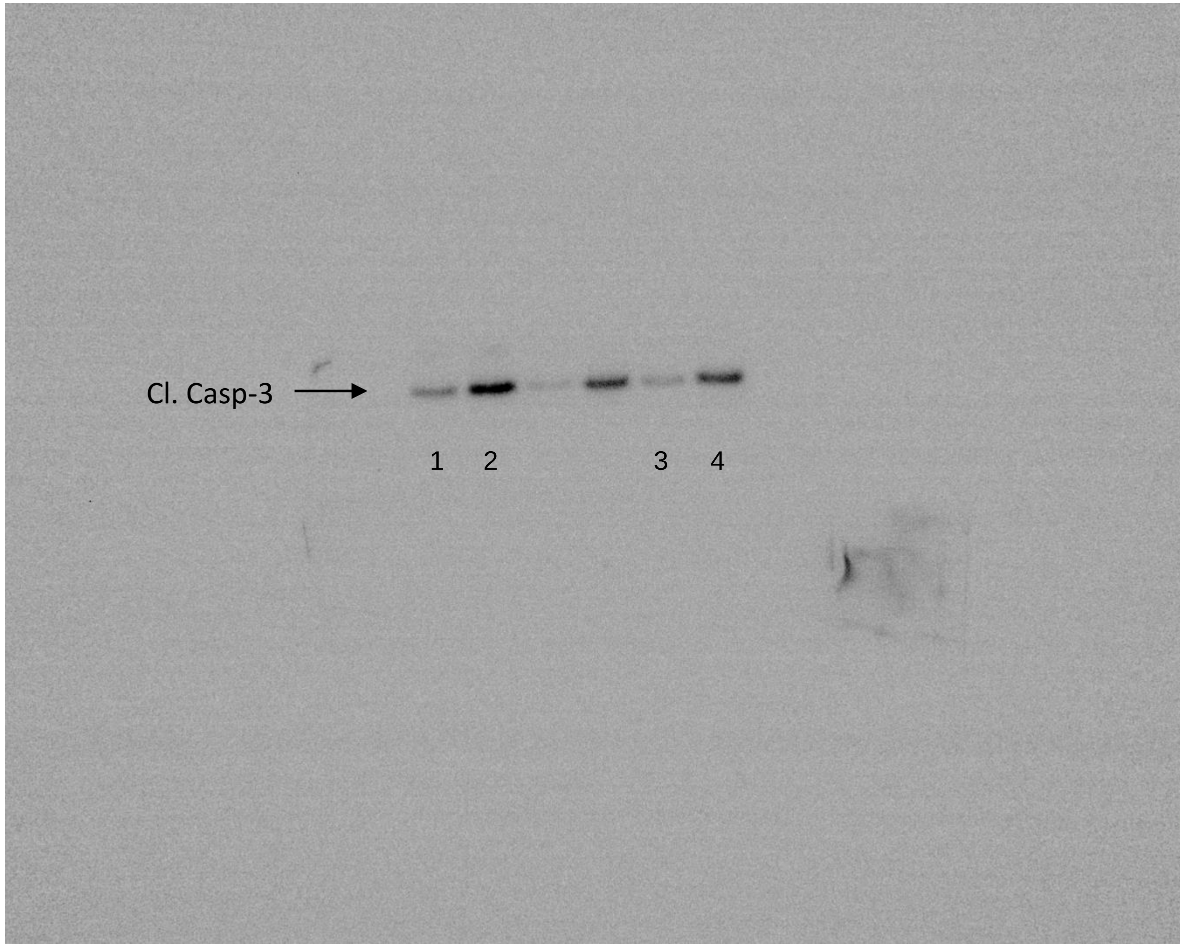

Full and uncropped western blot for Figure 7A
Lanes 1, 2, 3, 4 are on the figure

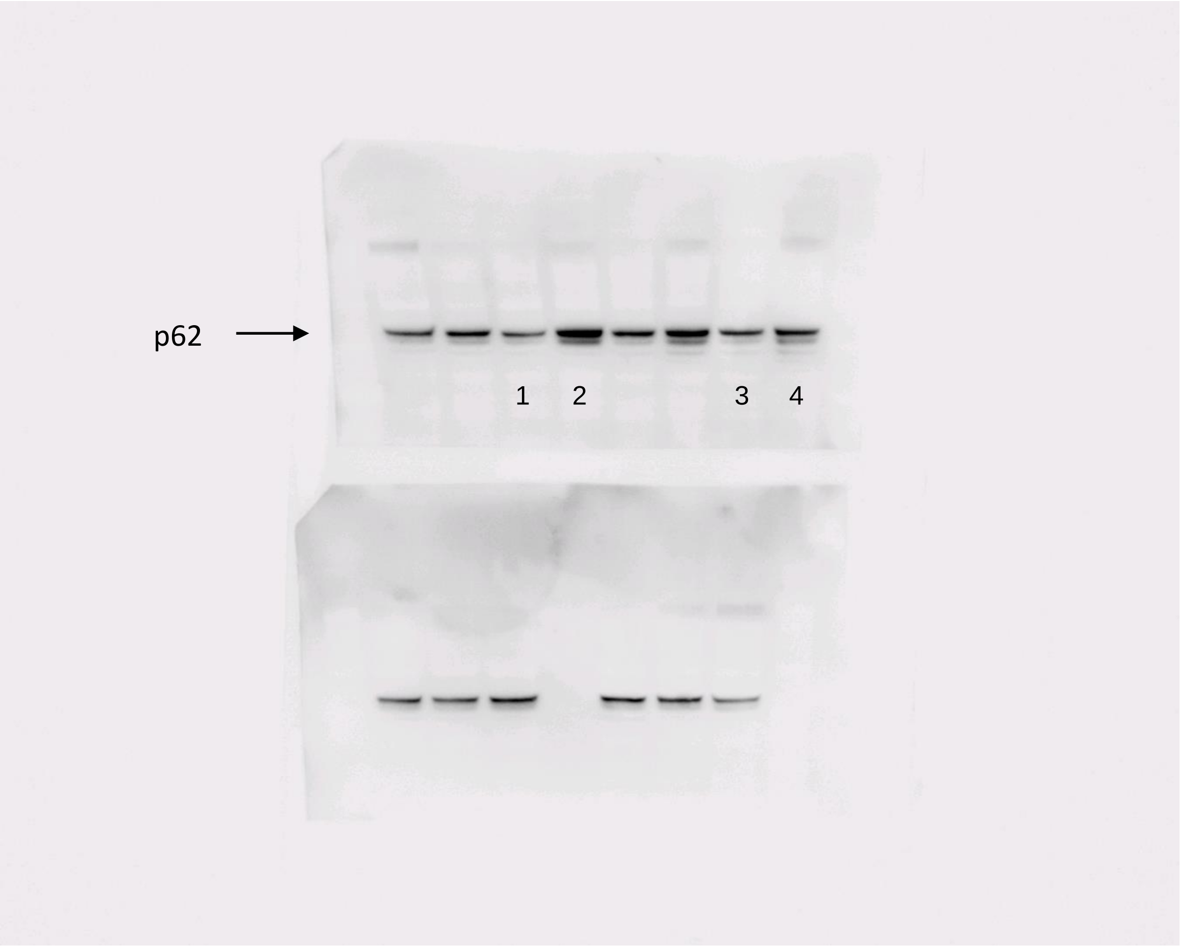

Full and uncropped western blot for Figure 7A

Lanes 1, 2, 3, 4 are on the figure

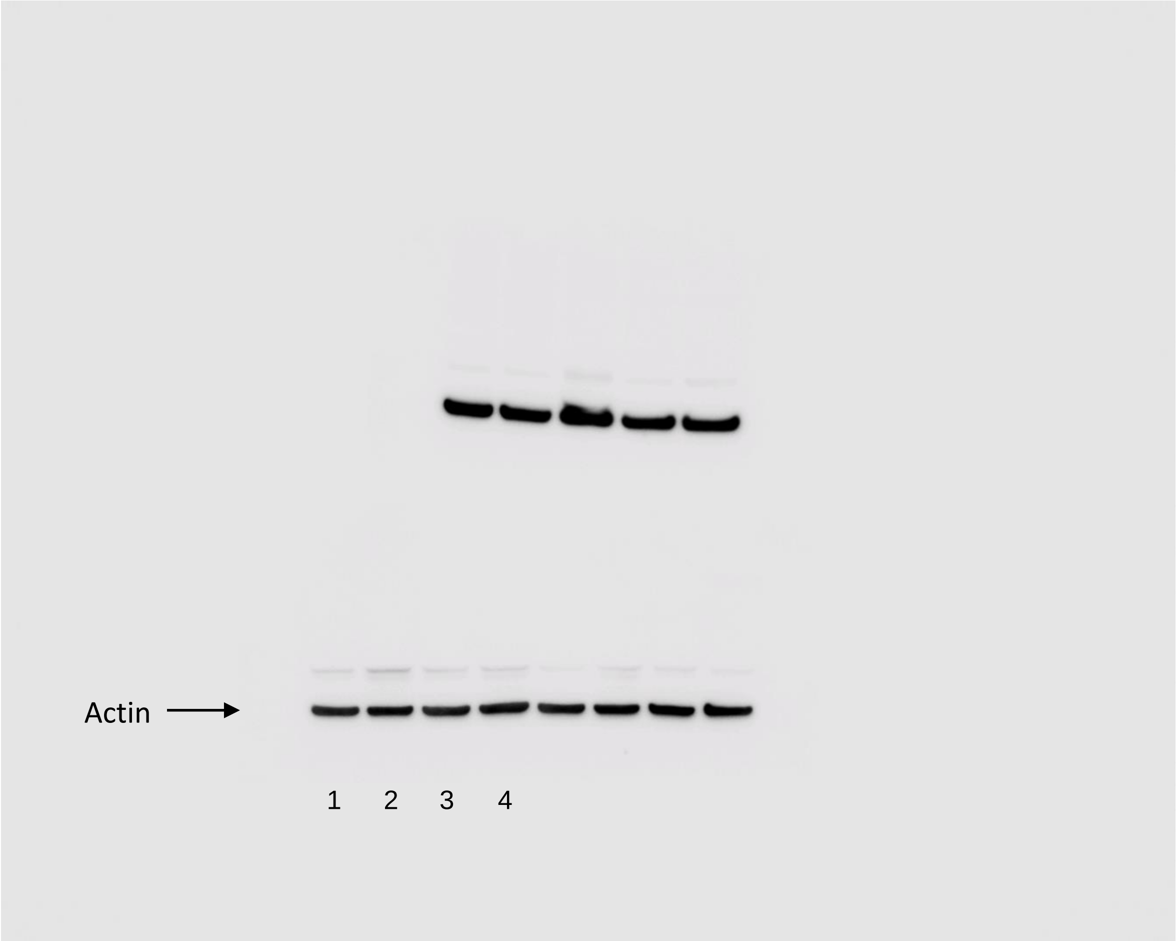

Full and uncropped western blot for Figure 7B
Lanes 1, 2, 3, 4 are on the figure

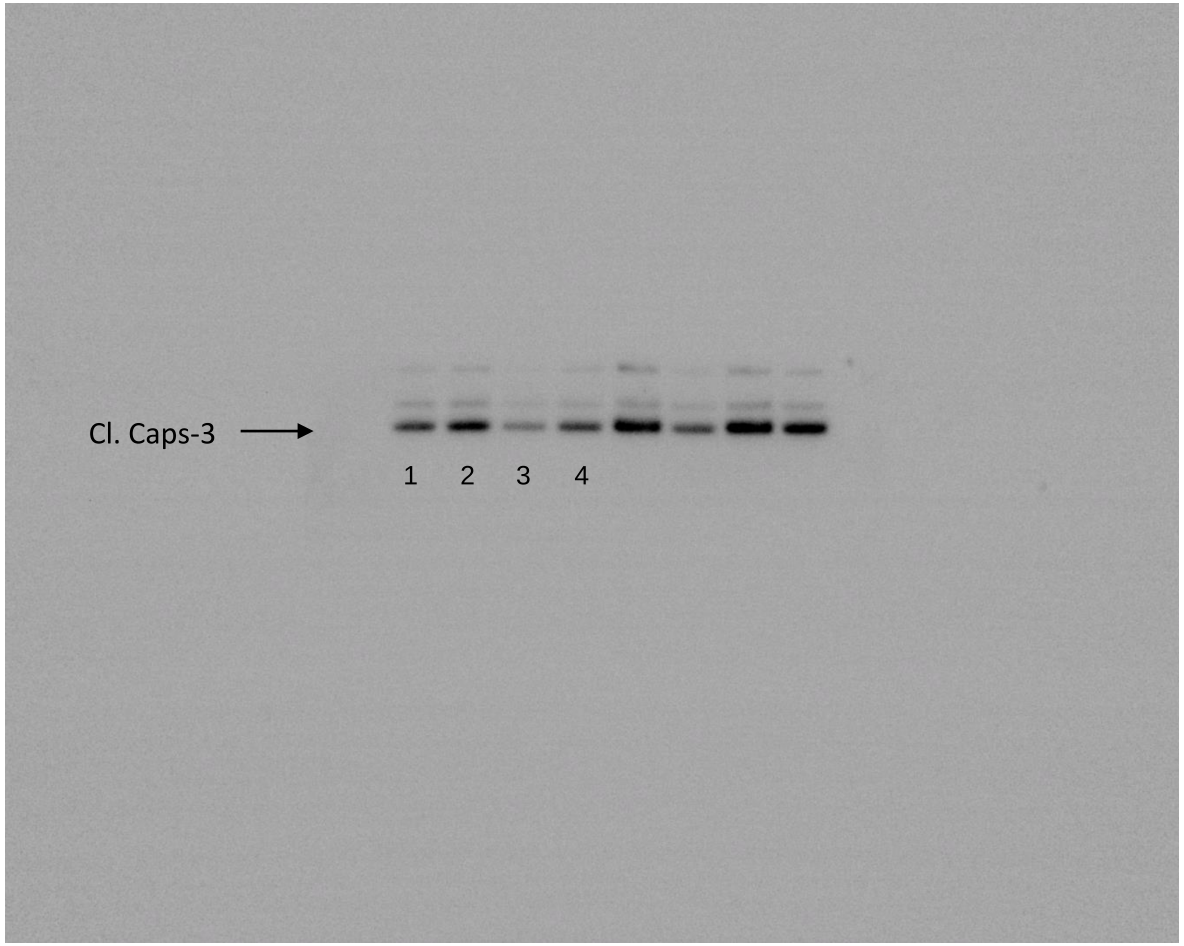

Full and uncropped western blot for Figure 7B

Lanes 1, 2, 3, 4 are on the figure

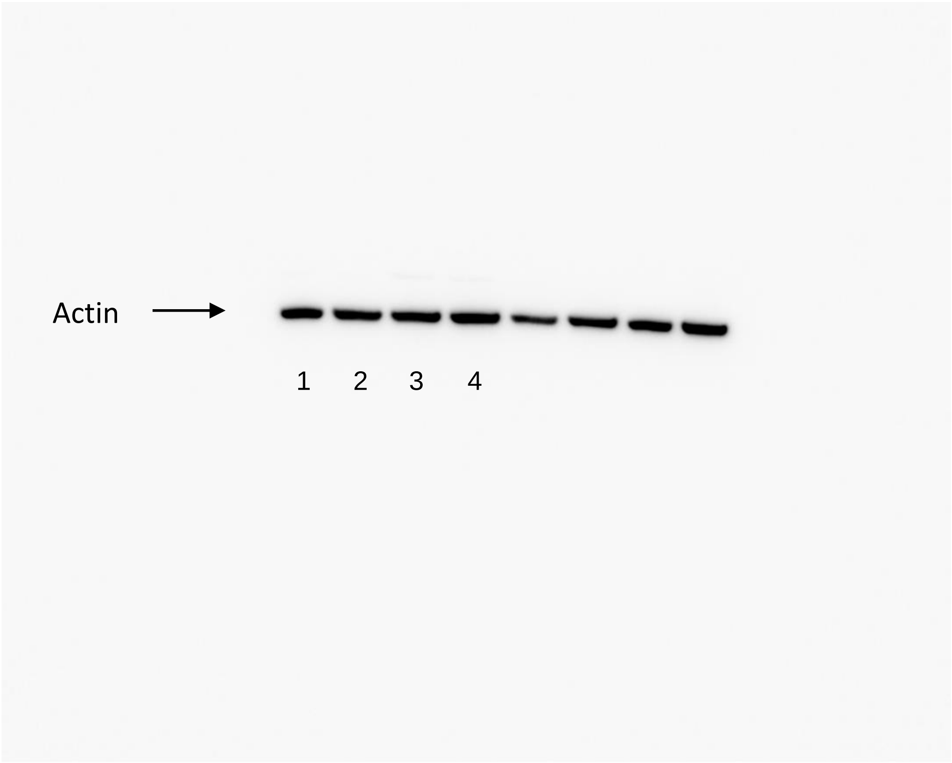

Full and uncropped western blot for Figure 7C

Lanes 1, 2, 3, 4 are on the figure

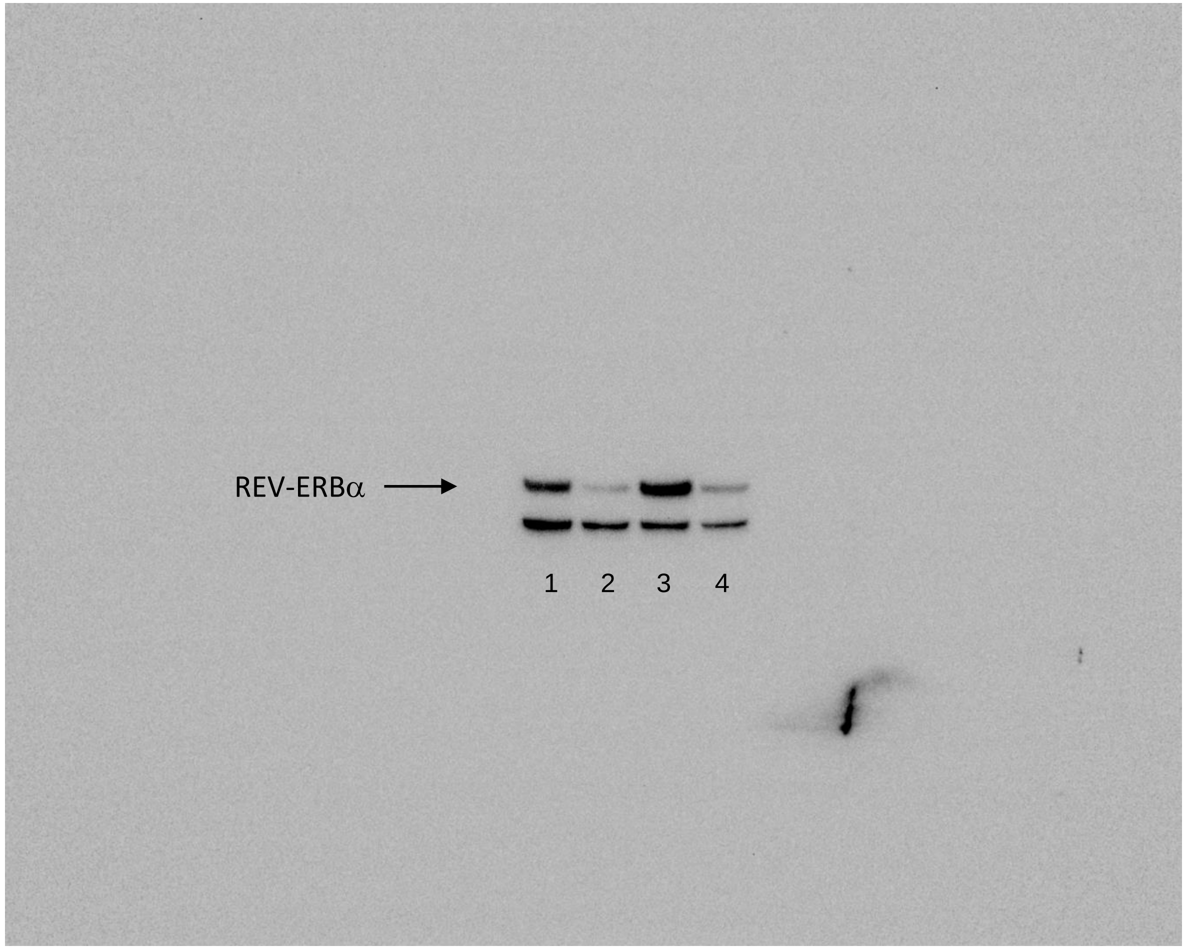

Full and uncropped western blot for Figure 7C
Lanes 1, 2, 3, 4 are on the figure

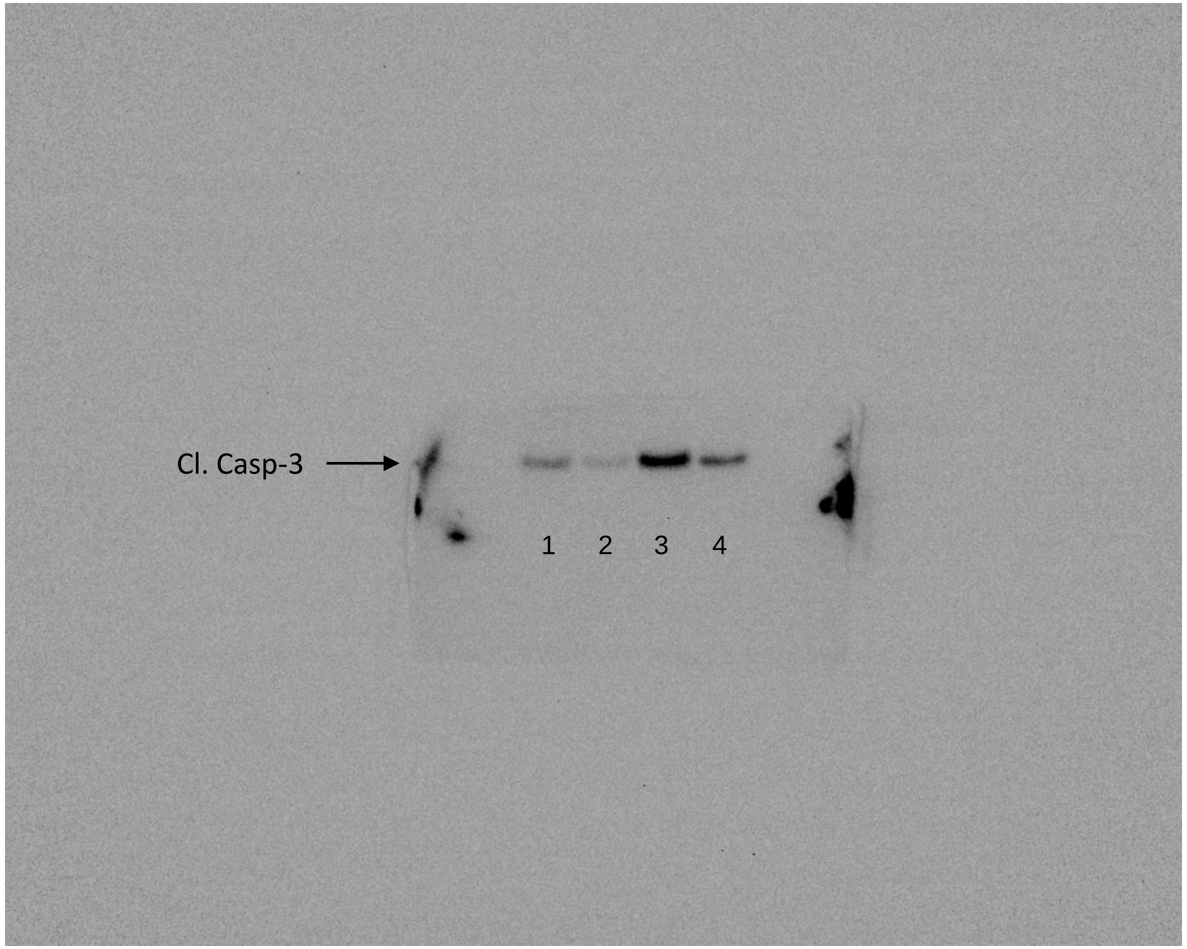

Full and uncropped western blot for Figure 7C

Lanes 1, 2, 3, 4 are on the figure

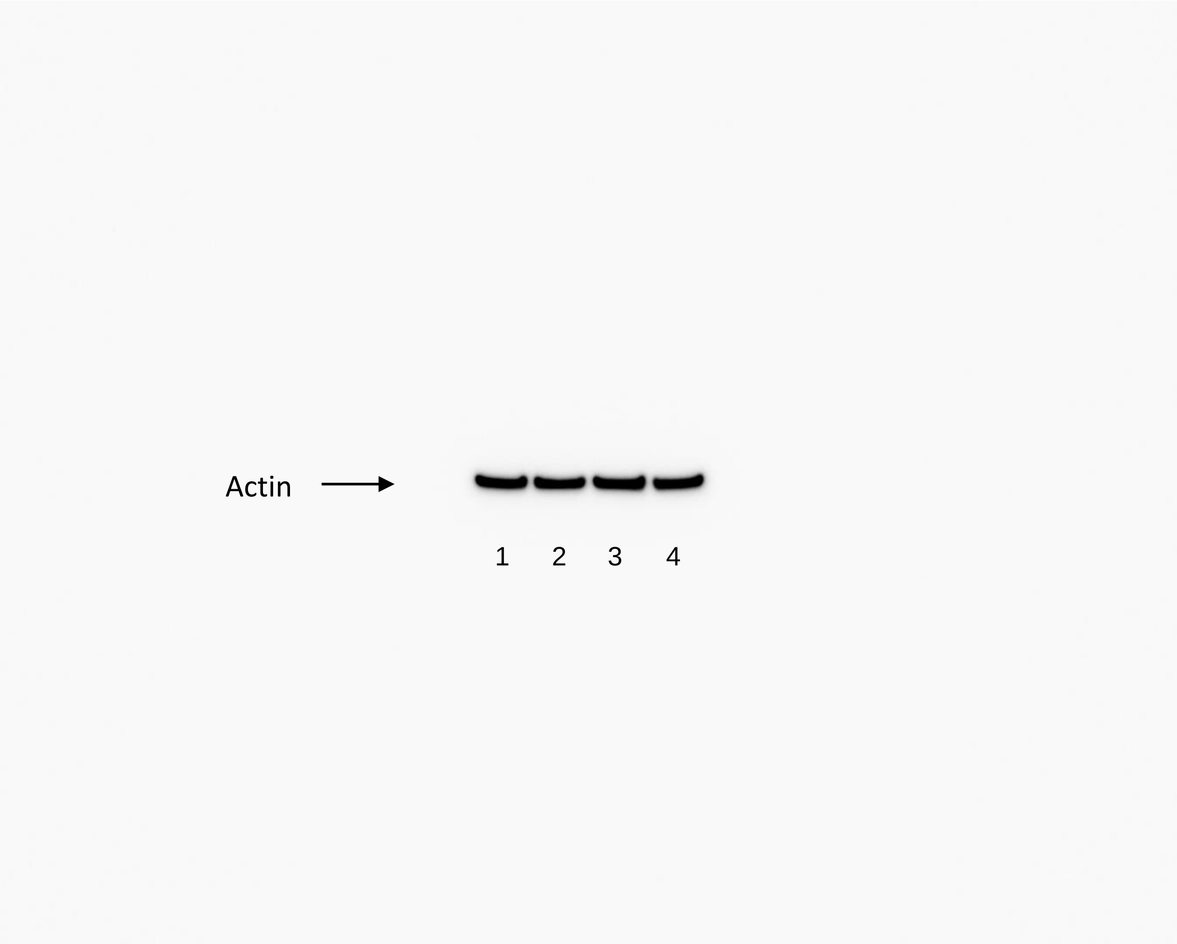

Supplement: Supplementary file 2 — Supplemental file_Uncropped WB [file 41419_2022_4767_MOESM2_ESM.pdf]
